# A simple and effective preparation of quercetin pentamethyl ether from quercetin

**DOI:** 10.3762/bjoc.14.291

**Published:** 2018-12-28

**Authors:** Jin Tatsuzaki, Tomohiko Ohwada, Yuko Otani, Reiko Inagi, Tsutomu Ishikawa

**Affiliations:** 1 Tokiwa Phytochemical Co. Ltd., 158 Kinoko, Sakura, Chiba 285-0801, Japan; 2 Graduate School of Pharmaceutical Sciences, The University of Tokyo, 7-3-1 Hongo, Bunkyo-ku, Tokyo 113-0013, Japan; 3 Graduate School of Medicine, The University of Tokyo, 7-3-1 Hongo, Bunkyo-ku, Tokyo 113-0013, Japan

**Keywords:** computational calculation, per-*O*-methylation, quercetin, quercetin pentamethyl ether, reactivity

## Abstract

Among the five hydroxy (OH) groups of quercetin (3,5,7,3',4'-pentahydroxyflavone), the OH group at 5 position is the most resistant to methylation due to its strong intramolecular hydrogen bonding with the carbonyl group at 4 position. Thus, it is generally difficult to synthesize the pentamethyl ether efficiently by conventional methylation. Here, we describe a simple and effective per-*O*-methylation of quercetin with dimethyl sulfate in potassium (or sodium) hydroxide/dimethyl sulfoxide at room temperature for about 2 hours, affording quercetin pentamethyl ether (QPE) quantitatively as a single product. When methyl iodide was used in place of dimethyl sulfate, the *C*-methylation product 6-methylquercetin pentamethyl ether was also formed. A computational study provided a rationale for the experimental results.

## Introduction

**Figure 1:**
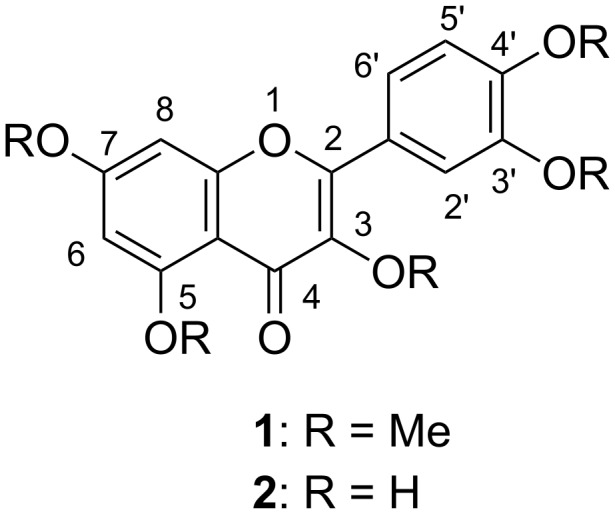
Structures of quercetin pentamethyl ether (QPE, **1**) and quercetin (**2**).

Flavonoids are distributed widely in plants, and exhibit various biological activities [1]. Polymethoxyflavones (PMFs) in particular have attracted much attention due to their broad spectrum of activities [2–8]. *Kaempferia parviflora* Wall. ex. Baker (Zingiberaceae), called “black turmeric” or “black ginger” in southern Asia, has been used as a folk medicine for vitality, as a nutritional supplement, and to reduce blood glucose levels; it has been reported to exhibit multiple biological activities [9–19]. Various PMFs have been isolated from this plant [13,19]. Recently our group reported that *K. parviflora* exhibited sirtuin-activating and antiglycation activities, and we showed that the active principles were PMFs, among which quercetin pentamethyl ether (QPE, 3,5,7,3',4'-pentamethylquercetin; 3,5,7,3',4'-pentamethoxyflavone, **1**, Figure 1) was the most potent [20]. Previous reports have shown that QPE (**1**) exhibits anticardiac hypertrophy [21], antidiabetic [22–23], antimetabolic disorder [13,24–25], antithrombotic [26] and α-glycosidase inhibition activities [14]. However, purification of large amounts of PMFs from natural sources for further studies of their bioactivities is difficult because of the presence of many structurally related compounds [2]. Therefore, simple and efficient syntheses are required.

PMFs can be synthesized from *o*-hydroxyacetophenone derivatives in several steps [27–28] or, more easily, from the corresponding phenolic flavones by conventional methylation, if the starting phenolic substrate is available [29–33]. Direct preparation of QPE (**1**) from quercetin (**2**) by per-*O*-methylation is difficult because the OH group at 5 position is resistant to alkylation due to its strong intramolecular hydrogen bonding with the carbonyl group at 4 position. Indeed, inconsistent experimental results have been reported in the literature; incomplete methylation occurred in some cases [24,34–36], though **1** was smoothly obtained in other cases [37–40]. Therefore, we set out to re-examine the per-*O*-methylation of **2** in detail. In this paper, we describe a simple, practical and effective preparation of **1** from quercetin (**2**). A computational study of the methylation reactions of **2** is also presented.

## Results and Discussion

### Reported per-*O*-methylation reactions and our re-examination

Conventional methylation of phenolic compounds is generally performed with methyl iodide (MeI) or dimethyl sulfate (Me_2_SO_4_) in an aprotic polar solvent in the presence of a base. Although acetone and potassium carbonate (K_2_CO_3_) are generally selected as the solvent and base, acetone can be replaced with dimethylformamide (DMF) if the starting phenol is poorly soluble in acetone.

To our knowledge four reactions [37–40] were found in the literature as successful per-*O*-methylations of quercetin (**2**), among which the three carried out methylations under the conventional conditions (Table 1). Using MeI and K_2_CO_3_ in DMF QPE (**1**) was isolated in 86% yield [37] (run 1 in Table 1). Per-*O*-methylations using acetone as a solvent have also been reported. Reactions under reflux with MeI and K_2_CO_3_ in 0.2 M solution [38] (run 2 in Table 1) and with Me_2_SO_4_ and K_2_CO_3_ in 0.023 M solution [39] (run 3 in Table 1) afforded **1** in 86% and 72% yields, respectively. In the remaining reaction the use of sodium hydride (NaH) as a base produced **1** in 84% yield [40] (run 4 in Table 1).

**Table 1:** Previous per-*O*-methylations of quercetin (**2**) and our trials using the reported conditions.

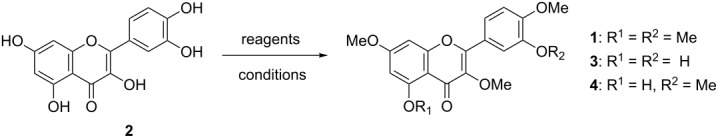								

run	methylating agent (equiv)	base (equiv)	solvent (mol/L)^a^	temp (°C)	time (h)	**1** (%)	reference	our results products (%)^b^

1	MeI (7)	K_2_CO_3_ (6)	DMF (0.4)	35	12	86	[37]	**1** (21), **3** (22), **4** (49)
2	MeI (7)	K_2_CO_3_ (3)	acetone (0.2)	reflux	24	86	[38]	**2** (86)
3	Me_2_SO_4_ (13)	K_2_CO_3_ (33)	acetone (0.023)	reflux	23	72	[39]	**1** (89)
4	MeI (16)	NaH (4)	DMF (0.05)	rt^c^	12	84	[40]	**3** (39)^d^

^a^**2**/solvent. ^b^Although commercially available **2** contains water, the yields of products were calculated based on the anhydrous form. The isolated yields are given. ^c^Room temperature. ^d^Tri-*O*-methyl derivative **3** was isolated as the main product, though multiple products including **1** and **4** were observed on TLC.

We at first re-examined these reactions under the reported conditions (Table 1, see Suporting Information, File1). In the first and last reactions, the desired QPE (**1**) was contaminated with 3,7,4'-trimethyl- (**3**) and 3,7,3',4'-tetramethylquercetin (**4**), albeit the main product was dependent upon the reaction conditions (runs 1 and 4 in Table 1). The reaction using a limited amount of acetone resulted in the recovery of the staring material due to its practical insolubility (run 2 in Table 1). On the other hand QPE (**1**) was obtained cleanly in good yield at high dilution, such that **2** was completely dissolved in the latter case (run 3 in Table 1). Thus, we found that an acceptable result was obtained only under the high dilution conditions using acetone as a solvent [39] among our re-examination trials. It is noteworthy that the per-*O*-methylated product **1** can be easily detected as a characteristic blue-fluorescent spot on a thin-layer chromatography (TLC) plate under irradiation with long wavelength UV light (360 nm).

#### Optimization of methylation conditions with Me_2_SO_4_

Based on our re-examination of the reported methods, we selected Me_2_SO_4_ as a methylating agent and performed the reaction with only a slight excess of reagents and a limited volume of solvent, to minimize the cost. The results are summarized in Table 2.

**Table 2:** Our studies on the per-*O*-methylation of quercetin (**2**) using Me_2_SO_4_.

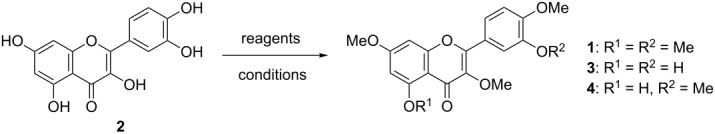							

run	Me_2_SO_4_ (equiv)	base (equiv)	catalyst (equiv)	solvent (mol/L)^a^	temp	time (h)	products (%)^b^

1^c^	6	10% NaOH aq (6)	Bn(Et)_3_NCl (0.1 )	CH_2_Cl_2_ (0.33)	rt^d^	4	**1** (31), **4** (46)
2	8	KOH (9)	none	DMSO (0.42)	rt^d^	2	**1** (85)
3^e^	9	NaOH (8)	none	DMSO (0.67)	rt^d^	2	**1** (66)
4	9	KOH (9)	none	DMF (0.84)	rt^d^	24	**1** (9), **3** (29), **4** (25)
5	9	K_2_CO_3_ (9)	none	DMSO (0.41)	rt^d^	24	a mixture (95%)^f^
6	5.5	K_2_CO_3_ (8)	none	DMF (0.5)	rt^d^	24	a mixture (91%)^g^

^a^**2**/solvent. ^b^Although commercially available **2** contains water, product yields were calculated based on the anhydrous form. Work-up was done by partition with EtOAc after quenching with H_2_O. The isolated yields are given. ^c^The use of larger amounts of reagents [Me_2_SO_4_ (8 equiv) and Bn(Et)_3_NCl (0.35 equiv)] decreased the combined yields of **1** and **4** (77%→68%); individual yields of **1** and **4** were 39% and 29% yields, respectively. ^d^Room temperature. ^e^Filtration was used for work-up. ^f^TLC revealed a mixture of **3**, **4** and an undefined product. The yield was calculated based on the weight balance of a crude product to **2**. ^g^TLC revealed a mixture of **1**, **3**, **4** and an undefined product. The yield was calculated based on the weight balance of a crude product to **2**.

Phase transfer catalysts (PTCs), such as quaternary ammonium salt, are effective catalysts for the alkylation of less reactive OH functions, such as a hydrogen-bonded phenolic group [41–42]. However, treatment of **2** with 6 equiv of Me_2_SO_4_ in a biphasic mixture of dichloromethane (CH_2_Cl_2_) and 10% sodium hydroxide (NaOH) aqueous solution in the presence of 0.1 equiv of benzyl(triethyl)ammonium chloride [Bn(Et)_3_NCl] as PTC at room temperature (rt) afforded a mixture of **4** and **1**, in which the desired **1** was a minor component (run 1 in Table 2, see Supporting Information File 1).

The tetramethyl ether **4** showed a lower-field-shifted signal at 12.64 ppm in the ^1^H NMR spectrum (see Supporting Information File 2), due to strong intramolecular hydrogen-bonding of the OH group at 5 position with the carbonyl group at 4 position. This indicates the difficult methylation of the OH group at 5 position. Therefore, we consider to apply the more strongly basic system of potassium hydroxide (KOH) and dimethyl sulfoxide (DMSO). To a suspension of powdered KOH (9 equiv) in DMSO were successively added quercetin (**2**) and Me_2_SO_4_ (8 equiv), and the resulting reaction mixture was stirred at rt (CAUTION: careful addition with control of temperature is necessary, because the reaction is exothermic). During the reaction the dark brownish mixture turned a lighter brown, and QPE (**1**) was precipitated. Complete methylation was confirmed by means of TLC (2 h), and **1** was easily isolated in pure form in 85% yield simply by partition with ethyl acetate (EtOAc, run 2 in Table 2). QPE (**1**) was also obtained when NaOH was used in place of KOH (run 3 in Table 2); in this case the product was isolated by filtration after quenching the reaction mixture with a large amount of water (ca. 9 volumes vs DMSO, see Supporting Information File 1). On the other hand, per-*O*-methylation was incomplete in the KOH/DMF, K_2_CO_3_/DMSO and K_2_CO_3_/DMF systems (runs 4, 5 and 6 in Table 2), suggesting that strongly basic potassium (or sodium) methylsulfinylmethylide (KCH_2_SOMe or NaCH_2_SOMe) [43–45] might be formed in the KOH (or NaOH)/DMSO system. However, almost no reaction was observed upon treatment of **2** with NaCH_2_SOMe prepared from DMSO and NaH (data not shown). This result suggests that the hydroxide anion may serve as an effective base in the per-*O*-methylation reaction of **2** using the KOH (or NaOH)/DMSO system.

#### Per-*O*-methylation trial with MeI

**Figure 2:**
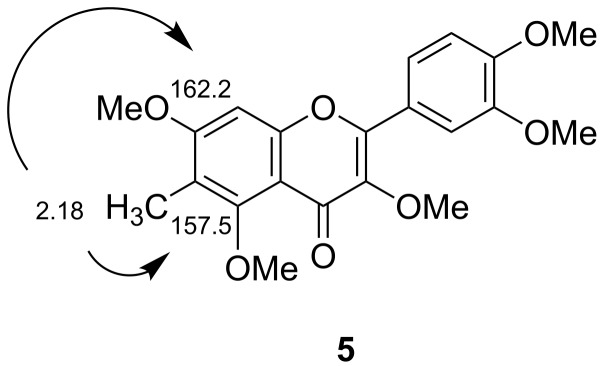
Selected HMBC correlations of 6-methyl-3,5,7,3',4'-penta-*O*-methylquercetin (**5**).

We next attempted per-*O*-methylation of **2** by use of MeI in place of Me_2_SO_4_ in the KOH/DMSO system (see Supporting Information File 1). Although smooth conversion of **2** to **1** (67%) occurred, a less polar component **5**, showing similar blue fluorescence to that of **1** under long-wavelength UV-light, was unexpectedly co-produced in a trace amount. The byproduct **5** was suggested to be a nuclear-methylated QPE derivative based on the appearance of a peak at *m*/*z*: 387 (MH^+^) in the MS and an additional 3H signal at 2.18 ppm due to a *C*-methyl group in the ^1^H NMR spectrum (see Supporting Information File 2). *C*-Methylation apparently occurred at the 6- or 8-position, since the *meta*-coupled aromatic signals of **1** at 6.33 and 6.49 ppm were replaced by a singlet signal at 6.69 ppm. Inspection of 2D NMR spectra data (COSY, NOESY, HSQC and HMBC, see Supporting Information File 2) confirmed that the byproduct **5** was 6-methyl-3,5,7,3',4'-pentamethylquercetin. Selected HMBC correlations between the *C*-methyl protons (2.18 ppm) and the root carbons (157.5 and 162.2 ppm) of 5- and 7-methoxyl groups are shown in Figure 2. This *C*-methylation product **5** had been yielded by the further methylation of 6-methyl-3,7,3',4'-tetra-*O*-methylquercetin, which was obtained in addition to QPE (**1**) and the tetramethyl ether **4** in the methylation of **2** using a large excess of MeI (29 equiv) and KOH (11 equiv) in methanol (MeOH) [46–47].

### Computational insights

Kim et al. [35] reported that the order of reactivity of the OH groups for alkylation of **2** was 7 > 4' > 3 > 3' > 5. Rao et al. [48] showed that methylation occurred in the order of 4' > 7 > 3 > 3' > 5, while Bouktaib et al. [49] assigned a reactivity order of 7 > 3 ≈ 4' for monomethylation with a limited amount of MeI. On the other hand, Shi et al. [34] obtained a mixture of 7,4'-dimethyl and 3,7,4'-trimethyl ethers **3** when **2** was treated with 3.5 equiv of MeI and a mixture of 3,7,4'-trimethyl **3**, 3,7,3',4'-tetramethyl **4** and pentamethyl ethers **1** with 5 equiv of MeI. The latter three-component mixture was also obtained in our re-examination of the reported methylations as mentioned above. Furthermore, they [34] had independently reported the preparation of 3'-methylquercetin from **2** by the following successive reactions: tribenzylation, selective methylation of the OH group at 3' position in the resulting 3,7,4'-tribenzylquercetin, and deprotection of the benzyl groups. These results allowed us to deduce the reactivity order of the five OH functions on **2** to be 7 ≈ 4' > 3 > 3' > 5, and the rationale was accessed by computational studies.

#### Conformation of quercetin and stability of its oxyanions

Computational studies were carried out by using the Gaussian 09 suite of programs [50]. The geometries of all compounds were fully optimized by using the B3LYP/6-311++G(2d,p) level. Harmonic vibrational frequency calculations characterized the optimized structures as ground minima. Bulk solvation effects (self-consistent reaction field, SCRF) were simulated by using the CPCM method in DMSO as a solvent. The zero-point vibrational energy corrections were done without scaling.

**Figure 3:**
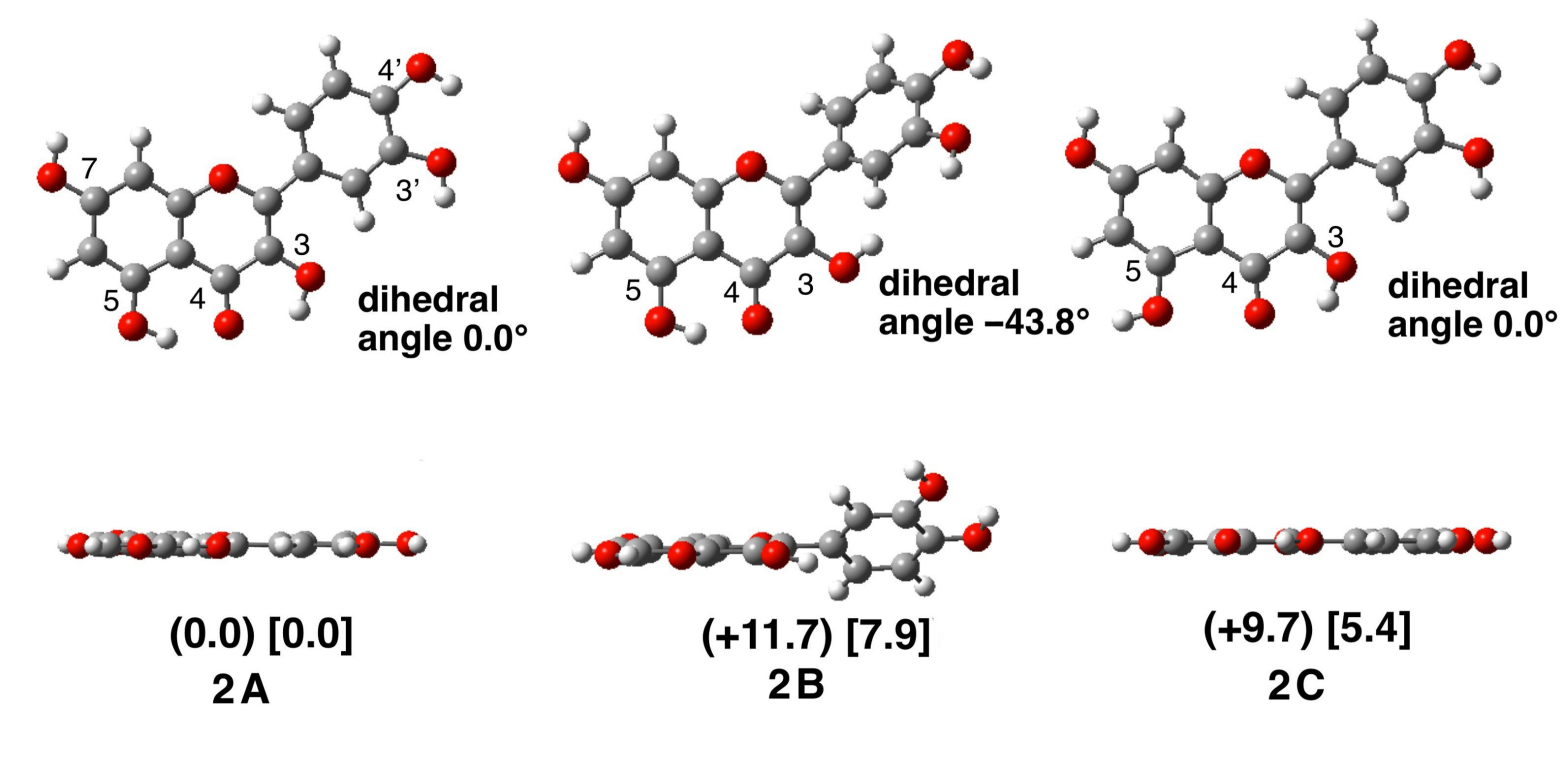
Three representative conformers of neutral quercetin (**2**). Top and side views are shown. The relative energy is shown in parentheses (gas phase) and in brackets (in DMSO, in kcal/mol). The dihedral angle around the biaryl structure (3–2–1'–2') is also shown.

Three typical conformers of quercetin (**2**) were considered (Figure 3). These are the regioisomers with respect to the direction of the OH groups at 3- and 5-positions. The most stable conformer **2A** contains a hydrogen bond between the OH group at 5 position and the carbonyl group at 4 position. The overall conformation is sensitive to the direction of the OH group at 3 position. The OH isomer at 3 position (**2B**) was significantly destabilized as compared with **2A**, by 11.7 kcal/mol (in the gas phase) and 7.9 kcal/mol (in DMSO). This is probably due to rotation of the 2-phenyl group with respect to the chromenone plane (by 44°). Furthermore, loss of hydrogen bonding of the OH group at 5 position with the carbonyl group at 4 position due to rotation of the OH group at 5 position destabilizes the system dramatically, as in **2C**, by 9.7 kcal/mol (in the gas phase) and 5.4 kcal/mol (in DMSO); however, the hydrogen bond is not crucial for the overall planar structure.

**Figure 4:**
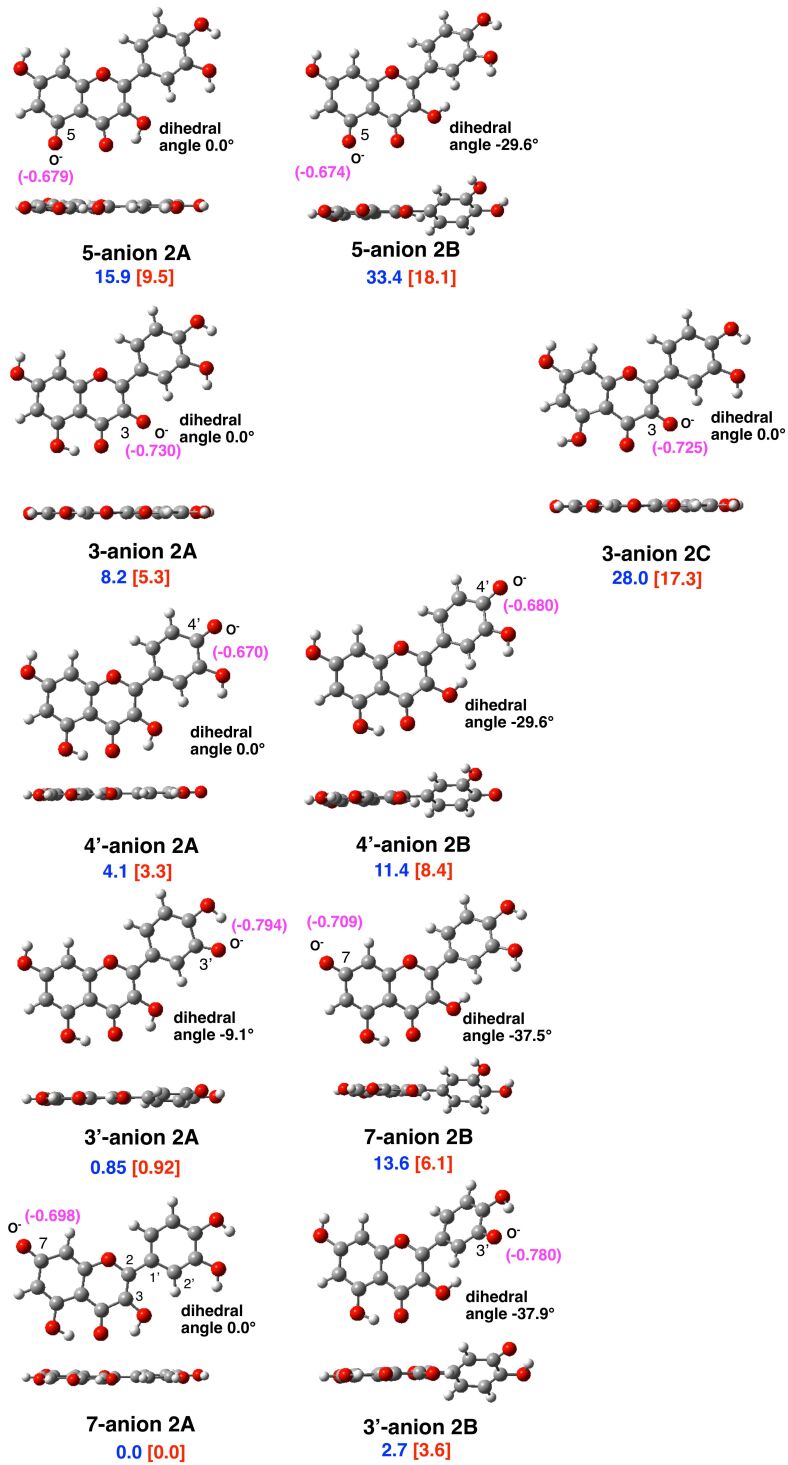
Structures of oxyanions derived from quercetin (**2**) by deprotonation of a single OH group. Top and side views are shown. The relative energy (in blue) is shown (gas phase) and in brackets (in red, in DMSO, in kcal/mol). The positions of the anions are based on the relative energies in DMSO. The dihedral angle around the biaryl structure (3–2–1'–2') is also shown. The natural bond orbital (NBO) charge of the anionic oxygen atom is shown in magenta.

The structures and energies of all possible isomeric mono-anions derived by deprotonation of a single OH group of quercetin (**2**) were calculated. The deprotonation is an endothermic process in this calculation model. The most stable anion was derived from the OH group at 7 position (**7-anion 2A**), which is stabilized by the conjugation with the carbonyl group at 4 position. The relative energies of all isomeric oxyanions are shown in Figure 4. All the monoanions (**anions 2A**) derived from the stable neutral conformer **2A** were more stable than those (**anions 2B**) from the conformer **2B**. Among the oxyanions at the OH group at 3 position (**3-anions**), we calculated the species (**3-anion 2C**) derived from the neutral conformation **2C**. The **3-anion 2C** was significantly more unstable than **3-anion 2A**, the geometrical difference being only the flip of the OH group at 5 position. This is consistent with a large stabilizing contribution of the hydrogen bond. The **5-anion 2A** was the most unstable species among the **anions 2A**, and the negative charge of the anionic oxygen is small (−0.679). While the thermodynamic stability (instability) and kinetic reactivity in methylation reaction do not always coincide, these features are consistent with the experimental finding that the OH group at 5 position shows the lowest reactivity, while the OH group at 7 position shows the highest reactivity. In the cases of **anions 2B**, all the corresponding anions **3'-anion 2B**, **4'-anion 2B**, **5-anion 2B**, **7-anion 2B** tended to take planar overall structures, that is, the biaryl dihedral angles (3–2–1'–2') were reduced from that of the neural species (**2B**: −43.8°). In particular, **4'-anion 2B** and **5-anion 2B** are affected by conjugation between the chromenone moiety and the phenyl group (biaryl dihedral angle: −29.6° in **4'-anion 2B**; −29.6° in **5-anion 2B**).

#### Change of proton affinity during the methylation reaction

**Figure 5:**
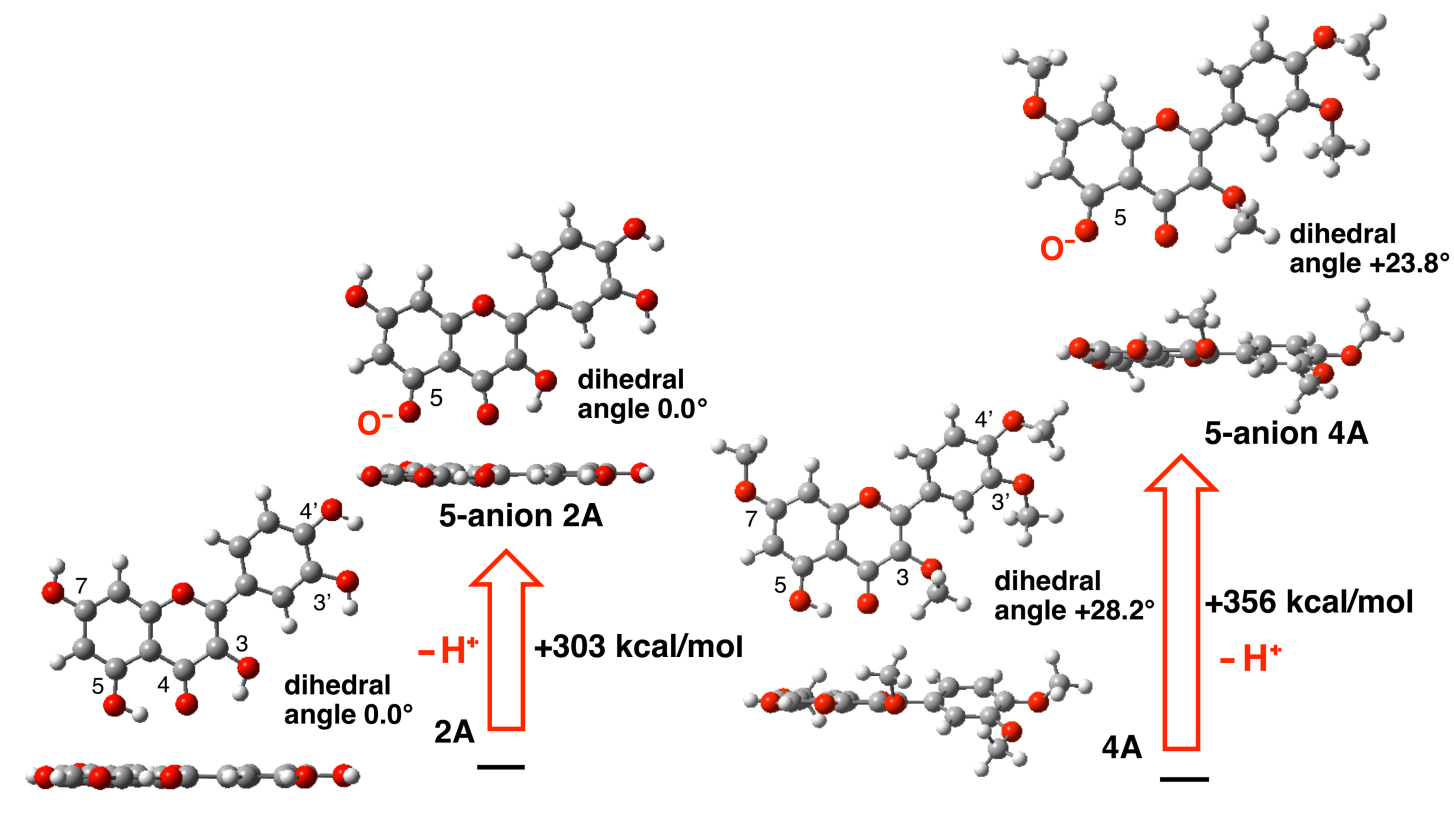
Deprotonation energy of the OH group at 5 position in neutral **2A** and tetra-*O*-methylated quercetin **4A**.

Although we focused on the deprotonation energy of quercetin (**2**), the deprotonation energy of each OH group may change with the progress of methylation. Figure 5 compares the deprotonation energies during generation of **5-anion 2A** from neutral quercetin **2A,** and **5-anion 4A** from neutral 3,7,3',4'-tetramethylquercetin (**4,** conformation **4A**). Conformation **4A** has a structure in which the phenyl group is rotated due to the OMe group at the 3 position (the biaryl dihedral angle is +28.2°), and upon ionization of the OH at 5 position to the oxyanion, the planarity was not significantly restored. This may increase the deprotonation energy in the case of neutral 3,7,3',4'-tetramethylquercetin (**4**). This result is consistent with the experimental observation of the difficulty of methylation of the OH group at 5 position, as exemplified by the formation of **4** in some of the per-*O*-methylation trials (see Table 2).

#### On *C*-methylation

**Figure 6:**
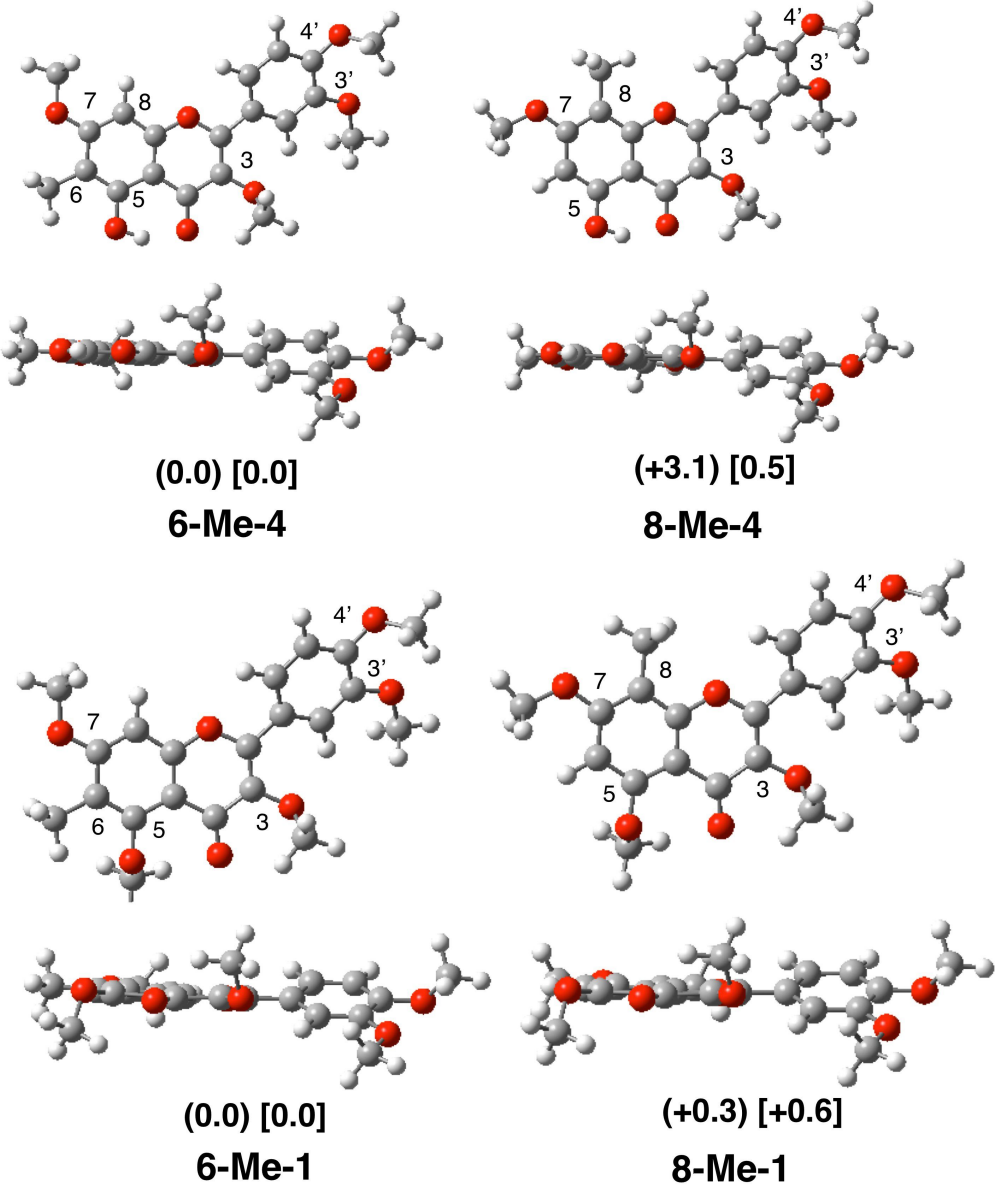
Structures and energies of *C*-methylated products of the tetra- **4** and the penta-*O*-methyl quercetins **1**.

In the methylation reaction using MeI, the *C*-methylation product was isolated as a minor product. On the basis of spectroscopic analysis, we identified this as the 6-methyl product rather than the 8-methyl product. The energy difference between the two isomeric *C*-methylation products of the tetramethyl ether **6-Me-4** and **8-Me-4** was 0.5 kcal/mol (in DMSO, Figure 6); the 6-methyl derivative **6-Me-4** was more stable than the 8-methyl one **8-Me-4**. The energy difference between the *C*-methylation isomers of per-*O*-methylated derivative **6-Me-1** and **8-Me-1** was of similar magnitude, 0.6 kcal/mol (in DMSO); again the 6-methylated product **6-Me-1** was more stable than the 8-methylated one **8-Me-1** (Figure 6). These calculation results are consistent with the structure **5** (= **6-Me-1**) obtained experimentally.

## Conclusion

In conclusion, a detailed re-examination of reported methods enabled us to develop a simple and effective per-*O*-methylation method of quercetin using an Me_2_SO_4_/KOH(or NaOH)/DMSO system as a cost–performance-optimized reaction. In addition, a computational study could provide a rationale for the experimental observations.

## Experimental

**General procedures.** Melting points were determined with a melting point hot-stage instrument without correction. ^1^H (400 MHz) and ^13^C NMR spectra (100 MHz) were recorded in CDCl_3_ on a Bruker Avance 400. Chemical shifts were shown in ppm (δ) values, and coupling constants are shown in hertz (Hz). Chemical shifts were calibrated with internal tetramethylsilane or with the solvent peak for the ^1^H and ^13^C NMR spectra. The following abbreviations are used: s = singlet, d = doublet, t = triplet, q = quartet, dd = double doublet. Electron spray ionization time-of-flight mass spectra (ESI–TOF MS) were recorded on a Bruker micrOTOF-05 to give high-resolution mass spectra (HRMS). Quercetin (**2**) was purchased from Tokyo Chemical Industry Co. Ltd., Japan.

**Methylation of quercetin (2) with the Me_2_SO_4_/KOH/DMSO system (run 2 in Table 2): QPE [2-(3,4-Dimethoxyphenyl)-3,5,7-trimethoxy-4*H*-1-benzopyran-4-one] (1).** To a suspension of powdered KOH (1.66 g, 29.7×10^−3^ mol) in DMSO (8 mL) was slowly added quercetin (**2**, 1 g, 3.3 × 10^−3^ mol), followed by Me_2_SO_4_ (2.5 mL, 26.4 × 10^−3^ mol), with control of the temperature to less than 10 °C (CAUTION: The reaction is exothermic). The resulting dark brown solution was stirred at rt for 2 h, during which time the color changed to light brown. The reaction was quenched with H_2_O (80 mL), and the resulting suspension was extracted with EtOAc (50/20/20 mL) The EtOAc solution was successively washed with 5% NaOH aq (10 mL × 4), H_2_O (10 mL × 3), and brine (10 mL), dried over sodium sulfate, and evaporated under reduced pressure to give QPE (**1**) as a light brown solid (1.05 g), which was homogeneous on TLC and showed a single peak at the same retention time as that of an authentic sample. Recrystallization from MeOH gave colorless prisms, mp 147–149 °C (lit. mp 136–137 °C [34]; mp 151 °C [39]; mp 151.2 °C [51]: ^1^H NMR δ 3.88 (s, 3H, OMe), 3.90 (s, 3H, OMe), 3.95 (s, 9H, OMe × 3), 6.34 (d, *J* = 2.2 Hz, 1H, 6- or 8-H), 6.49 (d, *J* = 2.2 Hz, 1H, 6- or 8-H), 6.97 (d, *J* = 8.4 Hz, 1H, 5'-H), 7.71 (dd, *J* = 8.4, 2.0 Hz, 1H, 6'-H), 7.72 (s-like, 1H, 2'-H); ^13^C NMR δ 55.9, 56.1, 56.3, 56.6, 60.1, 92.7, 96.0, 109.8, 111.2, 111.8, 121.8, 123.8, 141.4, 149.0, 151.2, 152.6, 159.0, 161.3, 164.1, 174.1.

## Supporting Information

File 1 Experimental procedures, ^1^H and ^13^C NMR assignments of methylated products **3**, **4** and **5** and references.

File 2 NMR charts of methylated products **1**, **3**, **4** and **5**.

## References

[1] Harborne J B, Williams C A (2000). Phytochemistry.

[2] Li S, Pan M-H, Wang Z, Lambros T, Ho C-T (2008). Tree and Forestry Science and Biotechnology.

[3] Li S, Pan M-H, Lai C-S, Lo C-Y, Dushenkov S, Ho C-T (2007). Bioorg Med Chem.

[4] Ho C-T, Pan M-H, Lai C-S, Li S (2012). J Food Drug Anal.

[5] Yuasa K, Tada K, Harita G, Fujimoto T, Tsukayama M, Tsuji A (2012). Biosci, Biotechnol, Biochem.

[6] Gosslau A, Chen K Y, Ho C-T, Li S (2014). Food Sci Hum Wellness.

[7] Matsuzaki K, Miyazaki K, Sakai S, Yawo H, Nakata N, Moriguchi S, Fukunaga K, Yokosuka A, Sashida Y, Mimaki Y (2008). Eur J Pharmacol.

[8] Ihara H, Yamamoto H, Ida T, Tsutsuki H, Sakamoto T, Fujita T, Okada T, Kozaki S (2012). Biosci, Biotechnol, Biochem.

[9] Tewtrakul S, Subhadhirasakul S (2007). J Ethnopharmacol.

[10] Wongsrikaew N, Kim H, Vichitphan K, Cho S K, Han J (2012). J Korean Soc Appl Biol Chem.

[11] Wattanapitayakul S K, Chularojmontri L, Herunsalee A, Charuchongkolwongse S, Chansuvanich N (2008). Fitoterapia.

[12] Sae-wong C, Tansakul P, Tewtrakul S (2009). J Ethnopharmacol.

[13] Horikawa T, Shimada T, Okabe Y, Kinoshita K, Koyama K, Miyamoto K-i, Ichinose K, Takahashi K, Aburada M (2012). Biol Pharm Bull.

[14] Azuma T, Kayano S-i, Matsumura Y, Konishi Y, Tanaka Y, Kikuzaki H (2011). Food Chem.

[15] Sawasdee P, Sabphon C, Sitthiwongwanit D, Kokpol U (2009). Phytother Res.

[16] Rujjanawate C, Kanjanapothi D, Amornlerdpison D, Pojanagaroon S (2005). J Ethnopharmacol.

[17] Wattanapitayakul S K, Suwatronnakorn M, Chularojmontri L, Herunsalee A, Niumsakul S, Charuchongkolwongse S, Chansuvanich N (2007). J Ethnopharmacol.

[18] Akase T, Shimada T, Terabayashi S, Ikeya Y, Sanada H, Aburada M (2011). J Nat Med.

[19] Shimada T, Horikawa T, Ikeya Y, Matsuo H, Kinoshita K, Taguchi T, Ichinose K, Takahashi K, Aburada M (2011). Fitoterapia.

[20] Nakata A, Koike Y, Matsui H, Shimada T, Aburada M, Yang J (2014). Nat Prod Commun.

[21] He T, Chen L, Chen Y, Han Y, Yang W-Q, Jin M-W (2012). Cardiovasc Drugs Ther.

[22] Wang Y, Xin X, Jin Z, Hu Y, Li X, Wu J, Jin M W (2013). Eur J Pharmacol.

[23] Xin X, Li X H, Wu J Z, Chen K H, Liu Y, Nie C J, Hu Y, Jin M W (2011). Eur J Pharmacol.

[24] Chen L, He T, Han Y, Sheng J-Z, Jin S, Jin M-W (2011). Molecules.

[25] Shen J Z, Ma L N, Han Y, Liu J X, Yang W Q, Chen L, Liu Y, Hu Y, Jin M W (2012). Diabetologia.

[26] Liang M-L, Da X-W, He A-D, Yao G-Q, Xie W, Liu G, Xiang J-Z, Ming Z-Y (2015). Sci Rep.

[27] Kawai S, Ikuina T, Hikima T, Tokiwano T, Yoshizawa Y (2012). Anticancer Res.

[28] Dao T T, Oh J W, Chi Y S, Kim H P, Sin K-S, Park H (2003). Arch Pharmacal Res.

[29] Liu Y, Xu X-H, Liu Z, Du X-L, Chen K-H, Xin X, Jin Z-D, Shen J-Z, Hu Y, Li G-R (2012). Biochem Pharmacol.

[30] Lee Y-J, Wu T-D (2001). J Chin Chem Soc.

[31] Dao T T, Kim S B, Sin K-S, Kim S, Kim H P, Park H (2004). Arch Pharmacal Res.

[32] Bernini R, Crisante F, Ginnasi M C (2011). Molecules.

[33] Mei Q, Wang C, Yuan W, Zhang G (2015). Beilstein J Org Chem.

[34] Shi Z-H, Li N-G, Tang Y-P, Wei-Li, Lian-Yin, Yang J-P, Hao-Tang, Duan J-A (2012). Eur J Med Chem.

[35] Kim M, Park Y, Cho S, Burapan S, Han J (2015). J Korean Soc Appl Biol Chem.

[36] Yuan J, Wong I L K, Jiang T, Wang S W, Liu T, Jin Wen B, Chow L M C, Wan Sheng B (2012). Eur J Med Chem.

[37] Pan G, Yang K, Ma Y, Zhao X, Lu K, Yu P (2015). Bull Korean Chem Soc.

[38] Juvale K, Stefan K, Wiese M (2013). Eur J Med Chem.

[39] Picq M, Prigent A F, Nemoz G, Andre A C, Pacheco H (1982). J Med Chem.

[40] Matsuda H, Morikawa T, Toguchida I, Yoshikawa M (2002). Chem Pharm Bull.

[41] Starks C M, Liotta C (1978). Phase Transfer Catalysis.

[42] Watanabe T, Ohashi Y, Yoshino R, Komano N, Eguchi M, Maruyama S, Ishikawa T (2003). Org Biomol Chem.

[43] Corey E J, Chaykovsky M (1965). J Am Chem Soc.

[44] Durst T (1969). Adv Org Chem.

[45] Martin D, Hauthal H G (1971). Dimethyl Sulphoxide.

[46] Jain A C, Seshadri T R (1953). J Sci Ind Res, Sect B.

[47] Jain A C, Seshadri T R (1954). J Sci Ind Res, Sect B.

[48] Rao K V, Owoyale J A (1976). J Heterocycl Chem.

[49] Bouktaib M, Lebrun S, Atmani A, Rolando C (2002). Tetrahedron.

[50] (2010). Gaussian 09.

[51] Sutthanut K, Sripanidkulchai B, Yenjai C, Jay M (2007). J Chromatogr A.

