# Supplementary material for: A simple and effective preparation of quercetin pentamethyl ether from quercetin
Source: Beilstein J Org Chem. 2018 Dec 28;14:3112–21. doi: 10.3762/bjoc.14.291 (PMC6317434; doi:10.3762/bjoc.14.291)
Supplement: File 1 — Experimental procedures, 1H and 13C NMR assignments of methylated products 3, 4 and 5 and references. [file Beilstein_J_Org_Chem-14-3112-s001.pdf]

BEILSTEIN JOURNAL OF ORGANIC CHEMISTRY

Supporting Information

for

A simple and effective preparation of quercetin pentamethyl ether from quercetin

Jin Tatsuzaki, Tomohiko Ohwada, Yuko Otani, Reiko Inagi and Tsutomu Ishikawa

Beilstein J. Org. Chem. **2018,** *14,* 3112–3121. doi:10.3762/bjoc.14.291

Experimental procedures, ^{1}H and ^{13}C NMR assignments of methylated products 3, 4 and 5 and references

License and Terms: This is a supporting information file under the terms of the Creative Commons Attribution License (http://creativecommons.org/licenses/by/4.0). Please note that the reuse, redistribution and reproduction in particular requires that the authors and source are credited.
The license is subject to the *Beilstein Journal of Organic Chemistry* terms and conditions: (https://www.beilstein-journals.org/bjoc)

General S2

Reaction with MeI/K_2CO_3/DMF (run 1 in Table 1) S2

Reaction with MeI/K_2CO_3/acetone (run 2 in Table 1) S2

Reaction with Me_2SO_4/K_2CO_3/acetone (run 3 in Table 1) S2

Reaction with MeI/NaH/DMF (run 4 in Table 1) S3

Reaction with Me_2SO_4/10% NaOH/CH_2Cl_2/Bn$(Et)_3$NCl (run 1 in Table 2) S3

Reaction with Me_2SO_4/NaOH/DMSO (run 3 in Table 2) S3

Reaction with MeI/KOH/DMSO S3

^{1}H and ^{13}C NMR assignments of quercetin trimethyl ether [5-hydroxy-2-(3-hydroxy-4-methoxyphenyl)-3,7-dimethoxy-4*H*-1-benzopyran-4-one] (**3**) S4

^{1}H and ^{13}C NMR assignments of quercetin tetramethyl ether [5-hydroxy-3,7-dimethoxy-2-(3,4-dimethoxyphenyl)-4*H*-1-benzopyran-4-one] (**4**) S4

^{1}H and ^{13}C NMR assignments of 6-methylquercetin pentamethyl ether [2-(3,4-dimethoxyphenyl)-3,5,7-trimethoxy-6-methyl-4*H*-1-benzopyran-4-one] (**5**) S4

References S5

General Melting points were determined with a melting point hot-stage instrument and are uncorrected. ESI mass spectra were obtained using a time-of-flight mass spectrometer. ^{1}H and ^{13}C NMR spectra were recorded at 400 and 100 MHz, respectively, in $CDCl_3$. Chemical shifts were calibrated with internal tetramethylsilane or with the solvent peak for the ^{1}H and ^{13}C NMR spectra. The following abbreviations are used: s = singlet, d = doublet, t = triplet, q = quartet, dd = double doublet, m = multiplet, and br = broad. For preparative TLC PLC Silica gel 60 F_{254} (1 or 2 mm) was used. Reaction mixture was extracted with ethyl acetate (EtOAc), and organic solutions obtained were washed with H_2O followed by brine and evaporated under reduced pressure after drying over $MgSO_4$, unless otherwise noted.

Reaction with MeI/K_2CO_3/DMF (run 1 in Table 1) According to the reported conditions [1] a suspension of quercetin (**2**) (0.146 g, 4.8 × 10^{-4} mol), K_2CO_3 (0.418 g, 30 × 10^{-4} mol), and MeI (0.5 g, 35 × 10^{-4} mol) in DMF (1.3 mL) was stirred at 35 °C for 24 h. After quenched with H_2O and acidified with 20% H_2SO_4 (pH ca 5) the mixture was extracted. Purification of the residue (0.157 g) by preparative TLC ($CHCl_3$) afforded quercetin tetramethyl ether **4** (Rf = 0.30-0.56, 0.084 g, 49%), quercetin trimethyl ether **3** (Rf = 0.12–0.24, 0.036 g, 22%), and quercetin pentamethyl ether **1** (Rf = 0.02–0.12, 0.039 g, 21%).

Reaction with MeI/K_2CO_3/acetone (run 2 in Table 1) According to the reported conditions [2] a suspension of **2** (0.156 g, 5.2 × 10^{-4} mol), K_2CO_3 (0.218 g, 15.8 × 10^{-4} mol), and MeI (0.5 g, 35 × 10^{-4} mol) in acetone (2.5 mL) was stirred under reflux for 24 h. After quenched with H_2O and acidified with 20% H_2SO_4 (pH ca 5) the mixture was centrifuged. The insoluble residue was washed with H_2O followed by acetone to recover **2** (0.134 g, 86%).

Reaction with Me_2SO_4/K_2CO_3/acetone (run 3 in Table 1) According to the reported conditions [3] a suspension of **2** (0.151 g, 5.0 × 10^{-4} mol), K_2CO_3 (2.3 g, 166 × 10^{-4} mol), and Me_2SO_4 (0.6 mL, 63 × 10^{-4} mol) in acetone (23 mL) was refluxed for 23 h under stirring. After addition of acetone the insoluble material was filtered off and washed with acetone. The

filtrate and the washings were combined and evaporated to give **1** (0.166 g, 89%), which was recrystallized from acetone.

Reaction with MeI/NaH/DMF (run 4 in Table 1) According to the reported conditions [4] a mixture of **2** (0.149 g, 4.9×10^{-4} mol), NaH (0.052 g, 22×10^{-4} mol), and MeI (0.5 mL, 80×10^{-4} mol) in DMF (10 mL) was stirred at rt for 21 h. After quenched with H_2O the mixture was extracted. Purification of the residue (0.166 g) by preparative TLC ($CHCl_3$: EtOAc = 8 : 1, v/v) afforded **3** (Rf = 0.34–0.65, 0.068 g, 39%) as a main product.

Reaction with Me_2SO_4/10% NaOH/CH_2Cl_2/Bn(Et)$_3$NCl (run 1 in Table 2) A mixture of **2** (0.50 g, 1.65×10^{-3} mol), Bn(Et)$_3$NCl (0.04 g, 0.18×10^{-3} mol), and Me_2SO_4 (0.94 mL, 9.9×10^{-3} mol) in CH_2Cl_2 (5 mL) and 10% NaOH aq solution (4 mL) was stirred at rt for 5 h. After quenched with H_2O and acidified with 20% H_2SO_4 (pH ca 5) the mixture was extracted. Purification of the residue (0.597 g) by preparative TLC ($CHCl_3$) afforded **4** (Rf = 0.12–0.24, 0.274 g, 46%) and **1** (Rf = 0.02–0.12, 0.193 g, 31%).

Reaction with Me_2SO_4/NaOH/DMSO (run 3 in Table 2) To a stirred suspension of powdered NaOH (0.22 g, 5.5×10^{-3} mol) in DMSO (1 mL) was slowly added **2** (0.20 g, 6.7×10^{-4} mol) followed by Me_2SO_4 (0.6 mL, 6.3×10^{-3} mol) at rt and the resulting mixture was stirred at rt for 2 h. After quenched with H_2O (10 mL) the insoluble material was filtered and washed with H_2O (to pH ca 7) to give **1** (0.17 g, 66%).

Reaction with MeI/KOH/DMSO To a stirred suspension of powdered KOH (0.83 g, 14.8 $\times 10^{-3}$ mol) in DMSO (4 mL) was slowly added **2** (0.51 g, 1.67×10^{-3} mol) followed by MeI (0.9 mL, 14.5×10^{-3} mol) at rt and the resulting suspension was stirred at rt for 1.5 h. After quenched with H_2O and acidified with 20% H_2SO_4 (pH ca 5) the mixture was extracted. The organic solutions were washed with 5% $NaHCO_3$ aq, H_2O and brine. Trituration of the residue (0.492 g) obtained with acetone afforded **1** (0.394 g) as off-white powder. Purification of the mother liquor by preparative TLC ($CHCl_3$/EtOAc = 5:1, v/v) afforded **5** (R_f = 0.34–0.48, 0.007 g, 1%) and an additional **1** (Rf = 0.14–0.28, 0.024 g; total 0.418 g, 67%).

On **5**: mp 158–160 °C; ESI–MS: *m/z* 387.1435 (calcd. for $C_{21}H_{23}O_7^+$: 387.1438). Anal. Calcd for $C_{21}H_{22}O_7$: C, 65.28; H, 5.74; Found: C, 65.25; H, 6.02.

^{1}H and ^{13}C NMR assignments of quercetin trimethyl ether [5-hydroxy-2-(3-hydroxy-4-methoxyphenyl)-3,7-dimethoxy-4*H*-1-benzopyran-4-one] (3) ^{1}H NMR (DMSO-d_6) δ 3.81 (3H, s, OMe), 3.867 (3H, s, OMe), 3.873 (3H, s, OMe), 6.37 (1H, d, *J* = 2.0 Hz, 6- or 8-H), 6.72 (1H, d, *J* = 2.0 Hz, 6- or 8-H), 7.11 (1H, d, *J* = 9.2 Hz, 5'-H), 7.58 (1H, dd, *J* = 9.2, 2.2 Hz, 6'-H), 7.59 (1H, d, *J* = 2.2 Hz, 2'-H), 9.44 (1H, s, OH), 12.65 (1H, s, OH); ^{13}C NMR (DMSO-d_6) δ 55.7, 56.1, 59.7, 92.3, 97.8, 105.2, 111.9, 115.1, 120.4, 122.2, 138.2, 146.4, 150.3, 155.6, 156.3, 160.9, 165.2, 178.1.

^{1}H and ^{13}C NMR assignments of quercetin tetramethyl ether [5-hydroxy-3,7-dimethoxy-2-(3,4-dimethoxyphenyl)-4*H*-1-benzopyran-4-one] (4) ^{1}H NMR δ 3.87 (3H, s, OMe), 3.88 (3H, s, OMe), 3.97 (3H, s, OMe), 3.98 (3H, s, OMe), 6.35 (1H, d, *J* = 2.4 Hz, 6- or 8-H), 6.44 (1H, d, *J* = 2.4 Hz, 6- or 8-H), 6.99 (1H, d, *J* = 8.4 Hz, 5'-H), 7.69 (1H, d, *J* = 2.4 Hz, 6'-H), 7.73 (1H, dd, *J* = 8.4, 2.4 Hz, 6'-H), 12.57-12.62 (1H, br, OH); ^{13}C NMR δ 56.0, 56.1, 56.2, 60.3, 92.4, 98.0, 106.2, 111.0, 111.5, 122.3, 123.1, 139.1, 148.9, 151.5, 156.0, 156.9, 162.2, 165.6, 178.9.

^{1}H and ^{13}C NMR assignments of 6-methylquercetin pentamethyl ether [2-(3,4-dimethoxyphenyl)-3,5,7-trimethoxy-6-methyl-4*H*-1-benzopyran-4-one] (5) ^{1}H NMR δ 2.18 (3H, s, CMe), 3.86 (3H, s, 3-OMe), 3.90 (3H, s, 5-OMe), 3.93 (3H, s, 7-OMe), 3.97 (3H, s, 3'-OMe), 3.97 (3H, s, 4'-OMe), 6.69 (1H, s, 8-H), 6.98 (1H, d, *J* = 8.8 Hz, 5'-H), 7.70-7.73 (2H, m, 2'- and 6'-H); ^{13}C NMR δ 8.1 (CMe), 55.9 (7-OMe), 56.0 (3'- or 4'-OMe), 59.9 (3'- or 4'-OMe), 61.5 (5-OMe), 94.6 (C8), 110.7 (C5'), 111.2 (C2'), 112.4 (C4a), 118.1 (C6), 121.6 (C6'), 123.4 (C1'), 141.0 (C3), 148.6 (C3'), 150.8 (C4'), 153.0 (C2), 156.4 (C8a), 157.5 (C5), 162.2 (C7), 173.4 (C4).

References

1. Pan, G.; Yang, K.; Ma, Y.; Zhao, X.; Lu, K.; Yu, P. *Bull. Korean Chem. Soc*. **2015**, *36*, 1460-1466.
2. Juvale, K.; Stefan, K.; Wiese, M. *Eur. J. Med. Chem*. **2013**, *67*, 115-126.
3. Picq, M.; Prigent, A. F.; Némoz, G.; André, A. C.; Pacheco, H. *J. Med. Chem*. **1982**, *25*, 1192-1198.
4. Matsuda, H.; Morikawa, T.; Toguchida, I.; Yoshikawa M. *Chem. Pharm. Bull*. **2002**, *50*, 788-795.
