# Supplementary material for: A simple and effective preparation of quercetin pentamethyl ether from quercetin
Source: Beilstein J Org Chem. 2018 Dec 28;14:3112–21. doi: 10.3762/bjoc.14.291 (PMC6317434; doi:10.3762/bjoc.14.291)

BEILSTEIN JOURNAL OF ORGANIC CHEMISTRY

Supporting Information

for

A simple and effective preparation of quercetin pentamethyl ether from quercetin

Jin Tatsuzaki, Tomohiko Ohwada, Yuko Otani, Reiko Inagi and Tsutomu Ishikawa

Beilstein J. Org. Chem. **2018,** *14,* 3112–3121. doi:10.3762/bjoc.14.291

NMR charts of methylated products 1, 3, 4 and 5

License and Terms: This is a supporting information file under the terms of the Creative Commons Attribution License (http://creativecommons.org/licenses/by/4.0). Please note that the reuse, redistribution and reproduction in particular requires that the authors and source are credited.
The license is subject to the *Beilstein Journal of Organic Chemistry* terms and conditions: (https://www.beilstein-journals.org/bjoc)

NMR charts of methylated products 1, 3, 4, and 5

Fig. S1. ^{1}H NMR (400 MHz, in $CDCl_3$) of quercetin pentamethyl ether **1** S2

Fig. S2. ^{13}C NMR (100 MHz, in $CDCl_3$) of quercetin pentamethyl ether **1** S3

Fig. S3. ^{1}H NMR (400 MHz, in DMSO-d_6) of quercetin trimethyl ether **3** S4

Fig. S4. ^{13}C NMR (100 MHz, in DMSO-d_6) of quercetin trimethyl ether **3** S5

Fig. S5. ^{1}H NMR (400 MHz, in $CDCl_3$) of quercetin tetramethyl ether **4** S6

Fig. S6. ^{13}C NMR (100 MHz, in $CDCl_3$) of quercetin tetramethyl ether **4** S7

Fig. S7. ^{1}H NMR (400 MHz, in $CDCl_3$) of 6-methylquercetin pentamethyl ether **5** S8

Fig. S8. ^{13}C NMR (100 MHz, in $CDCl_3$) of 6-metylquercetin pentamethyl ether **5** S9

Fig. S9. COSY of 6-methylquercetin pentamethyl ether **5** S10

Fig. S10. NOESY of 6-methylquercetin pentamethyl ether **5** S11

Fig. S11. HSQC of 6-methylquercetin pentamethyl ether **5** S12

Fig. S12. HMBC of 6-methylquercetin pentamethyl ether **5** S13

Fig. S1. ^{1}H NMR (400 MHz, in $CDCl_3$) of quercetin pentamethyl ether **1**

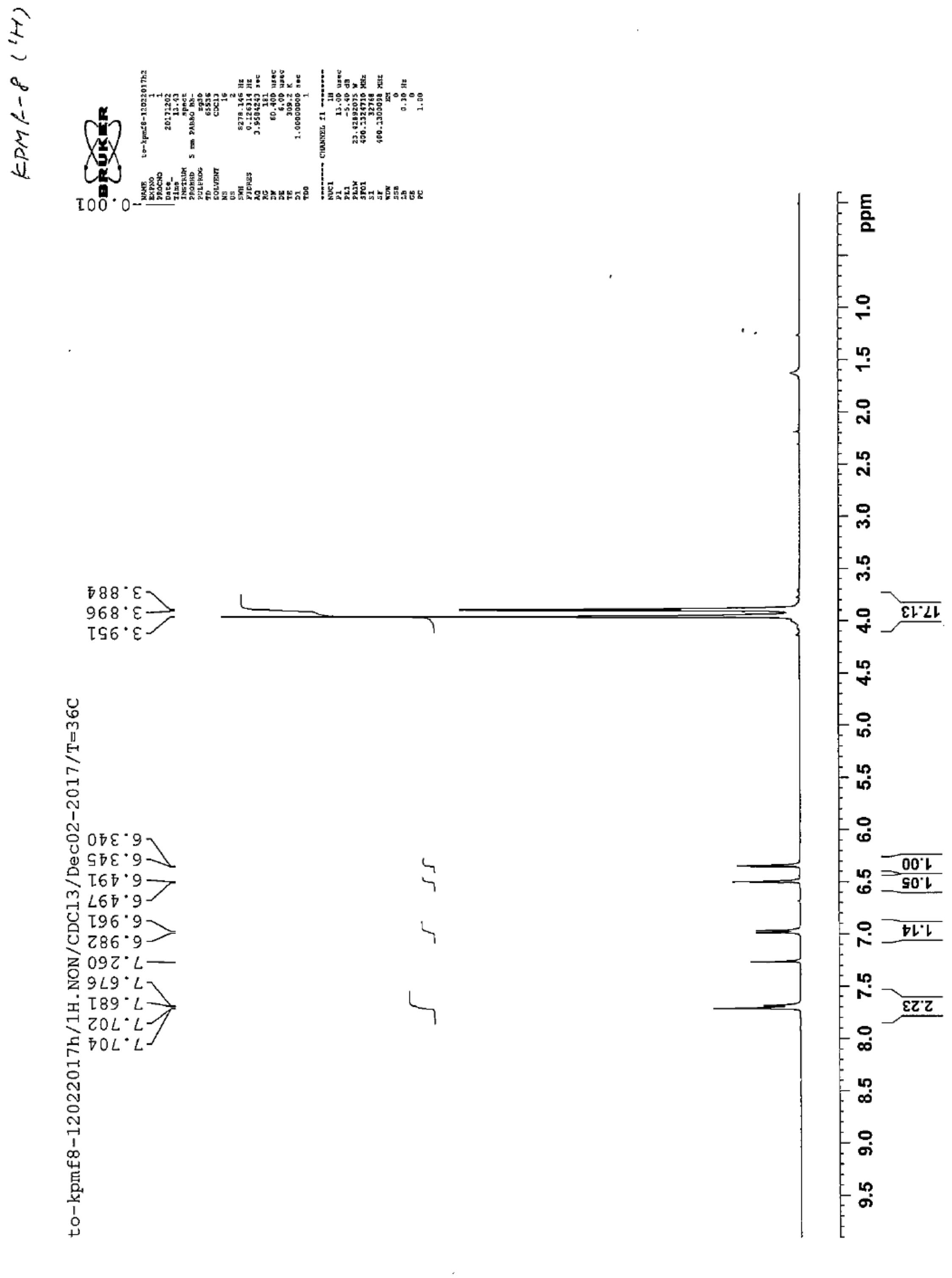

Fig. S2. ^{13}C NMR (100 MHz, in $CDCl_3$) of quercetin pentamethyl ether **1**

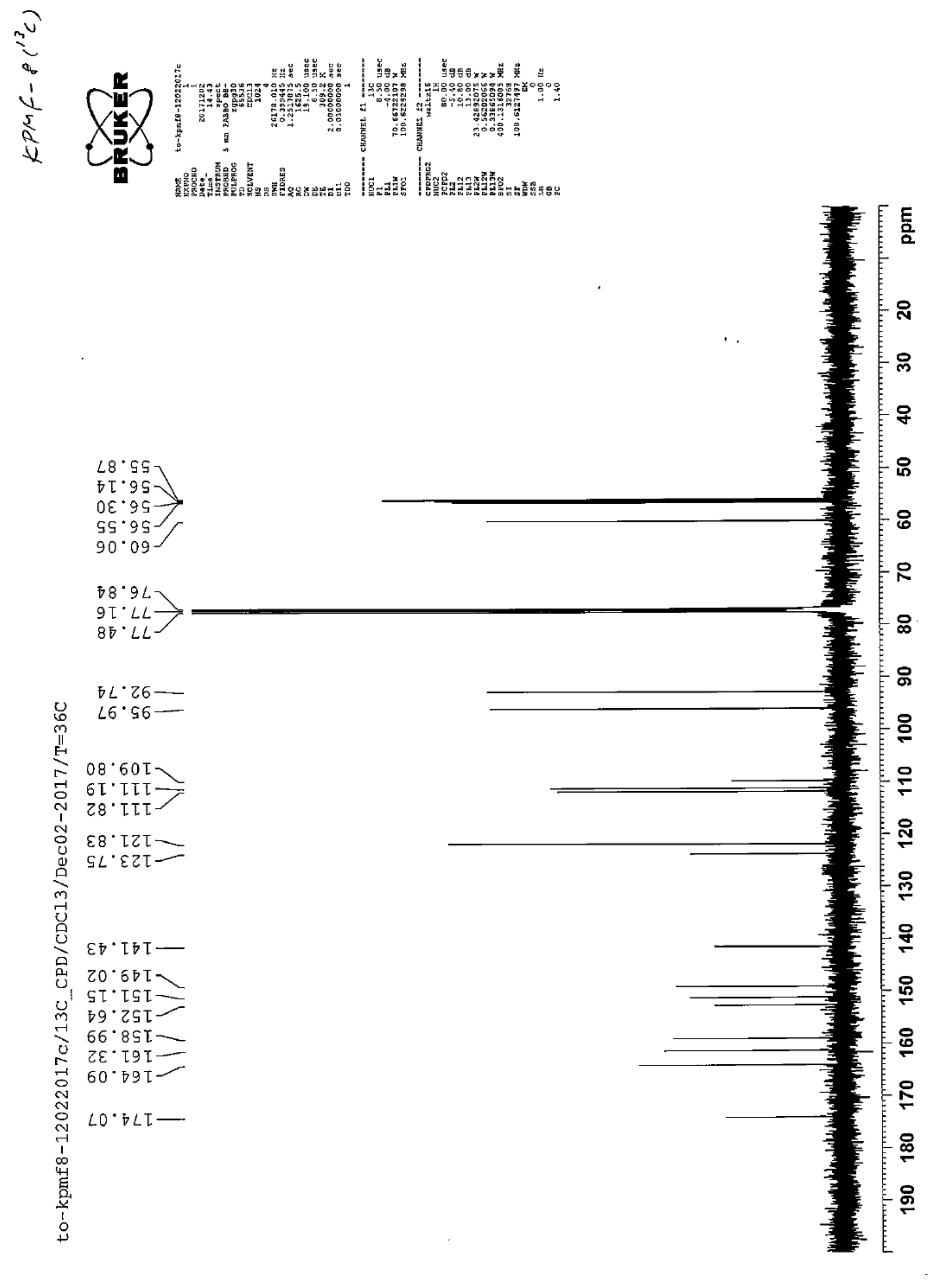

Fig. S3. ^{1}H NMR (400 MHz, in DMSO-d_6) of quercetin trimethyl ether **3**

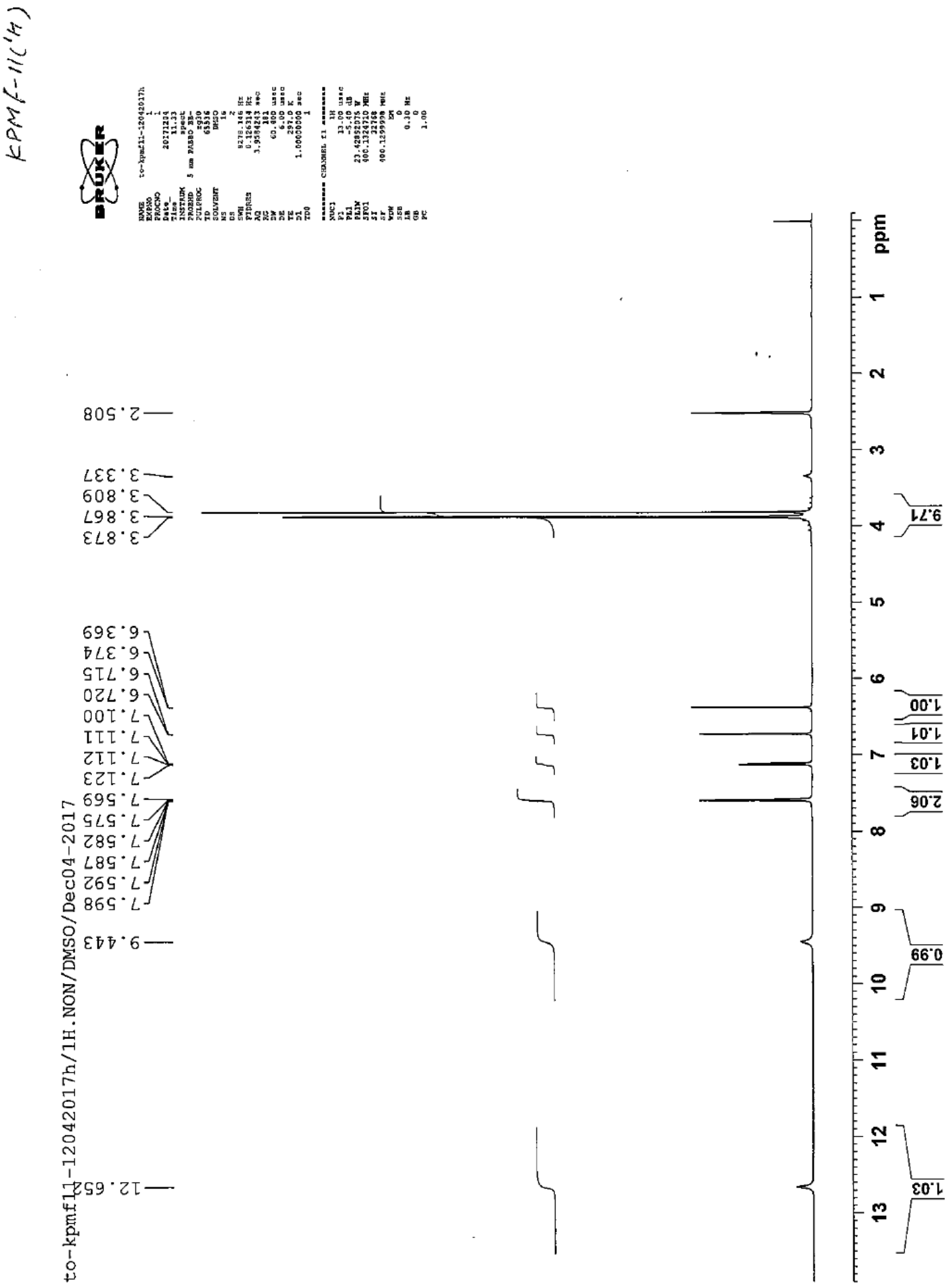

Fig. S4. ^{13}C NMR (100 MHz, in DMSO-d_6) of quercetin trimethyl ether **3**

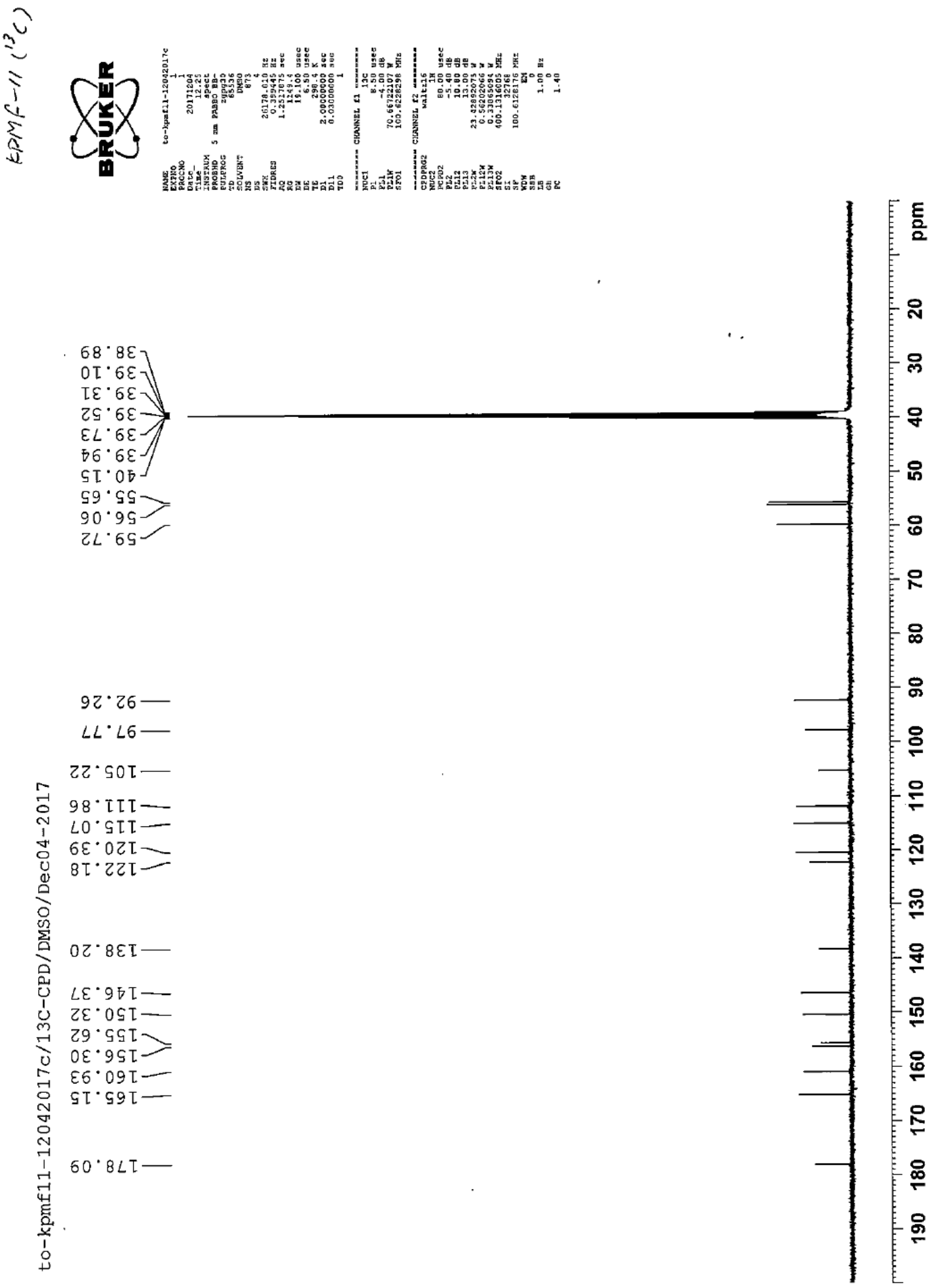

Fig. S5. ^{1}H NMR (400 MHz, in $CDCl_3$) of quercetin tetramethyl ether **4**

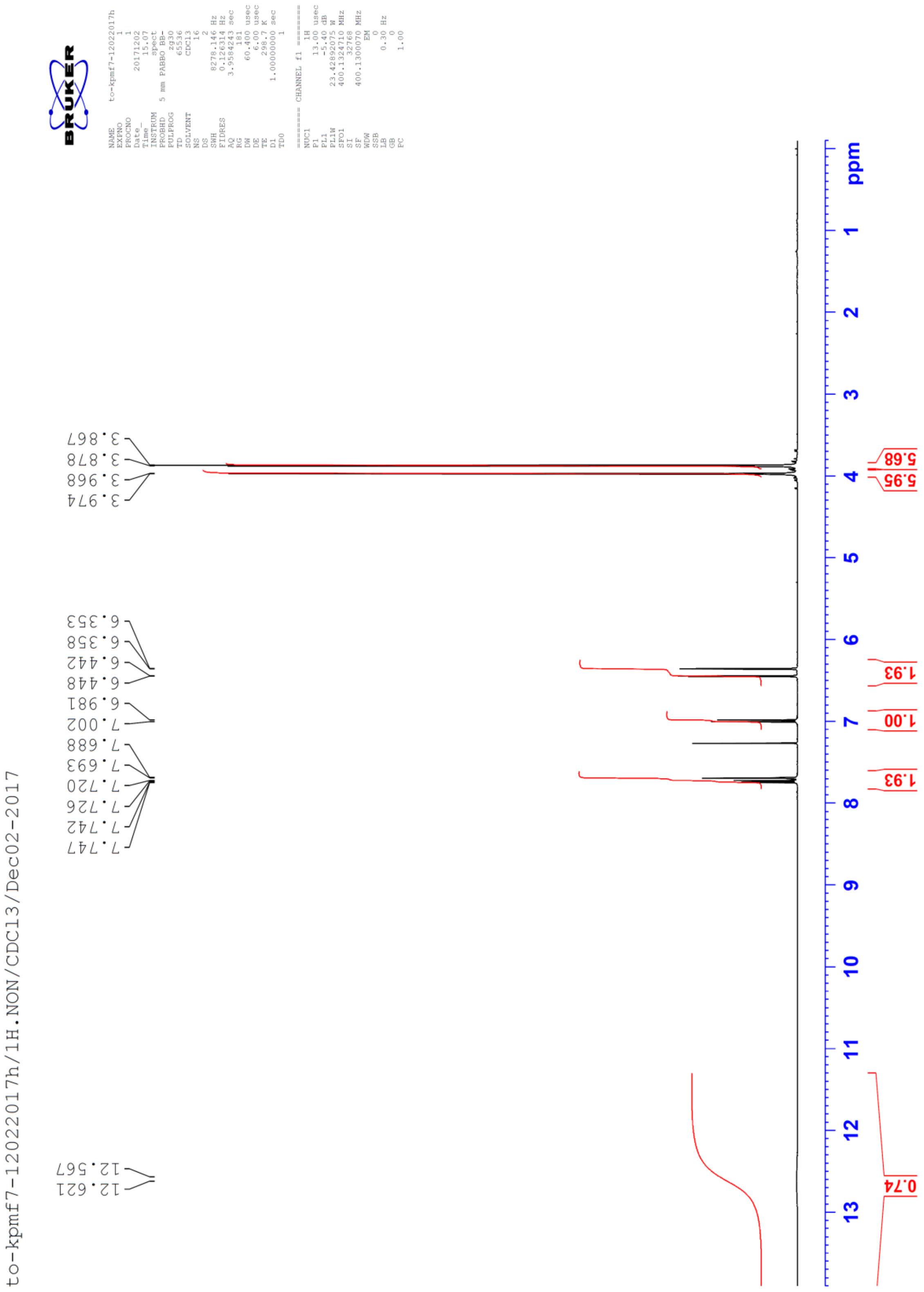

Fig. S6. ^{13}C NMR (100 MHz, in $CDCl_3$) of quercetin tetramethyl ether **4**

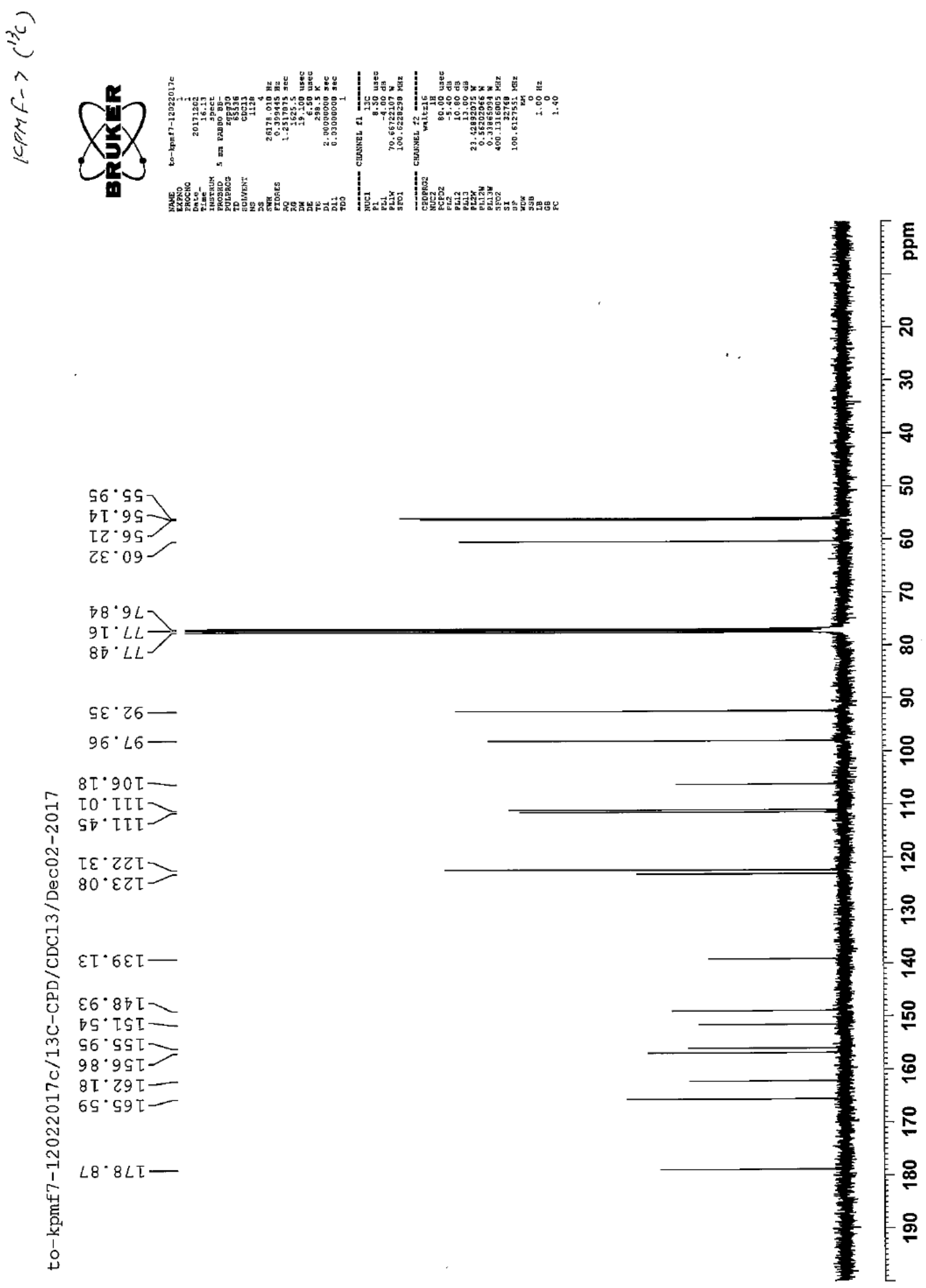

Fig. S7 ^{1}H NMR (400 MHz, in $CDCl_3$) of 6-methylquercetin pentamethyl ether **5**

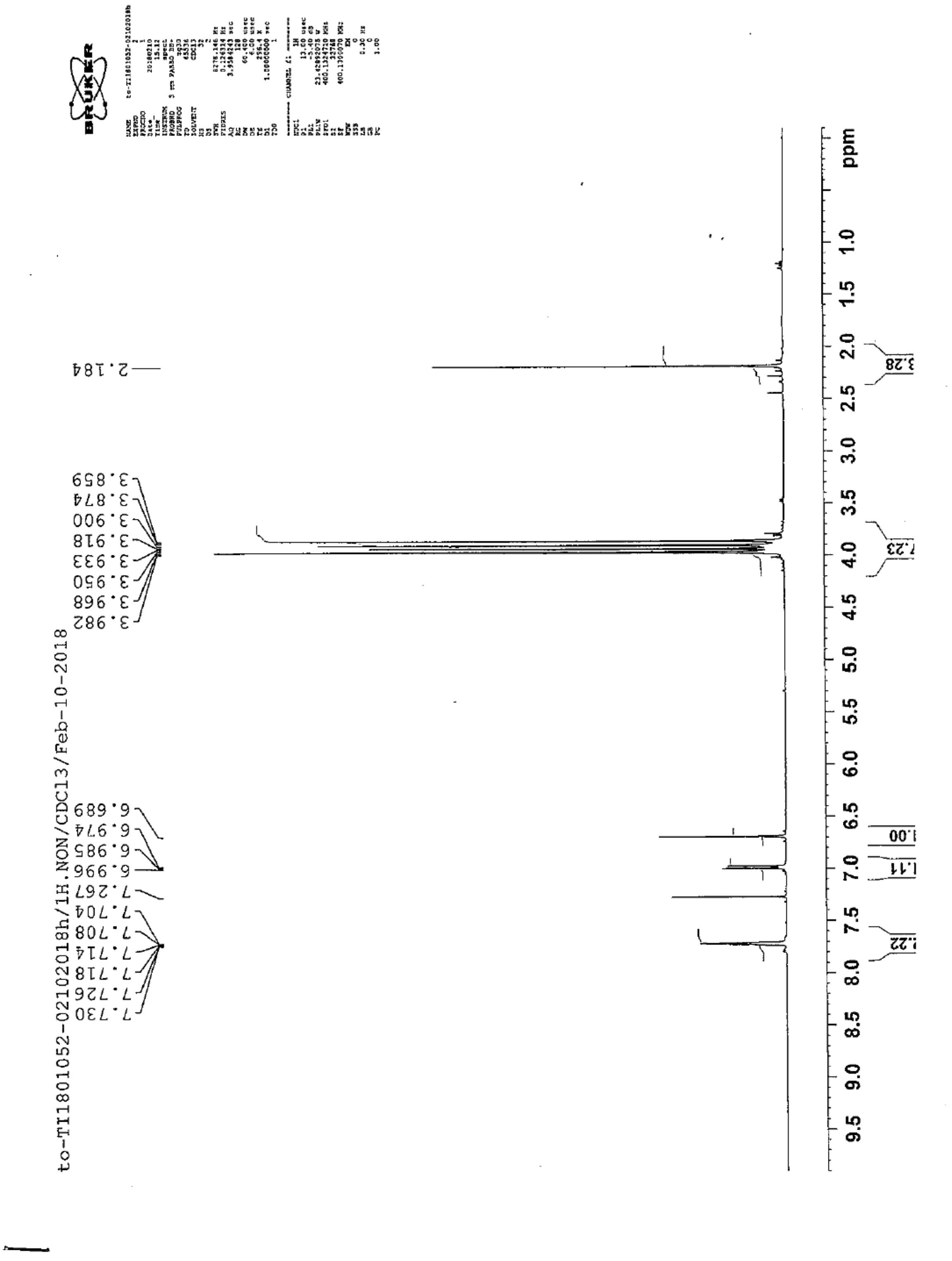

Fig. S8. ^{13}C NMR (100 MHz, in $CDCl_3$) of 6-methylquercetin pentamethyl ether **5**

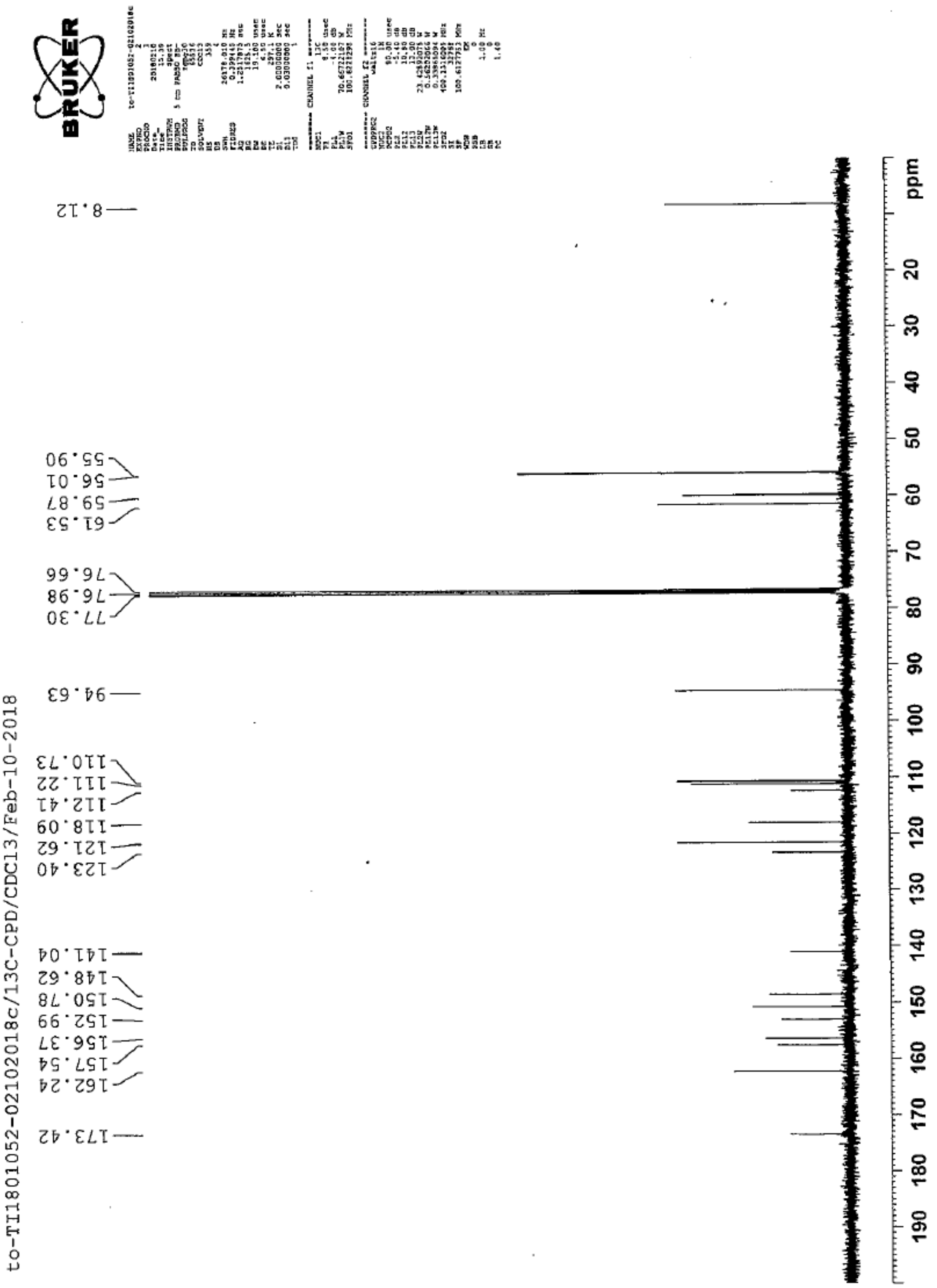

Fig. S9. COSY of 6-methylquercetin pentamethyl ether **5**

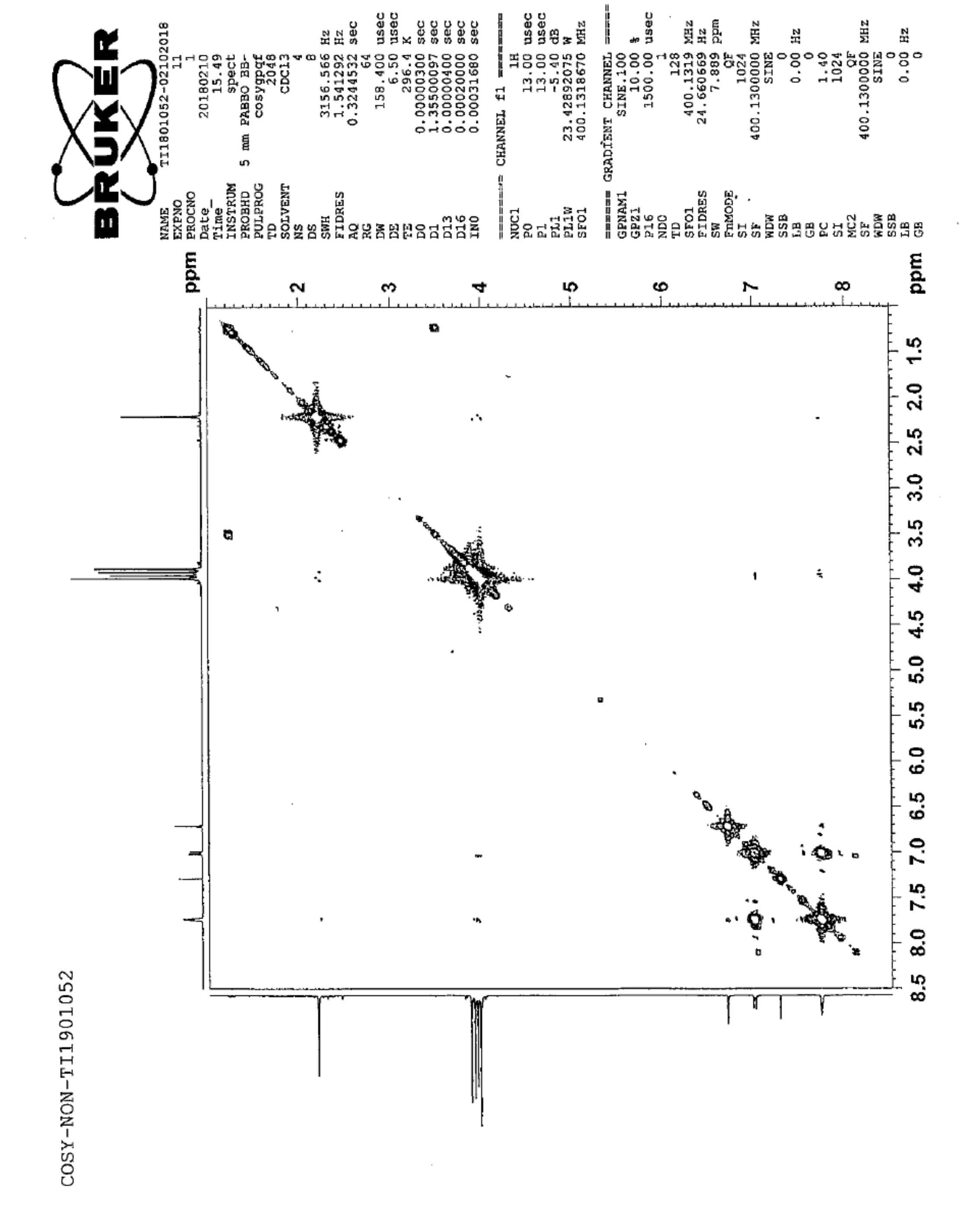

Fig. S10. NOESY of 6-methylquercetin pentamethyl ether **5**

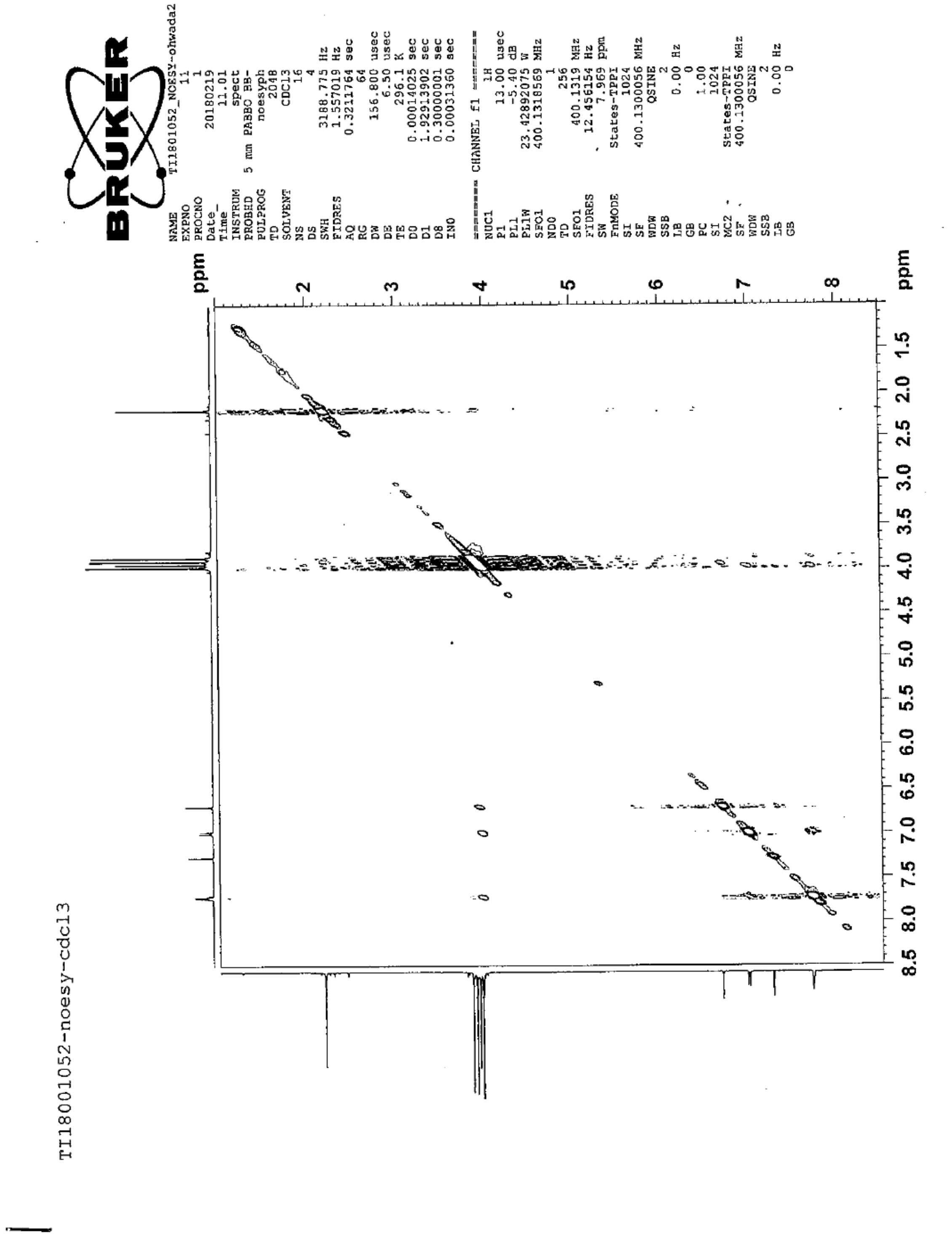

Fig. S11. HSQC of 6-methylquercetin pentamethyl ether **5**

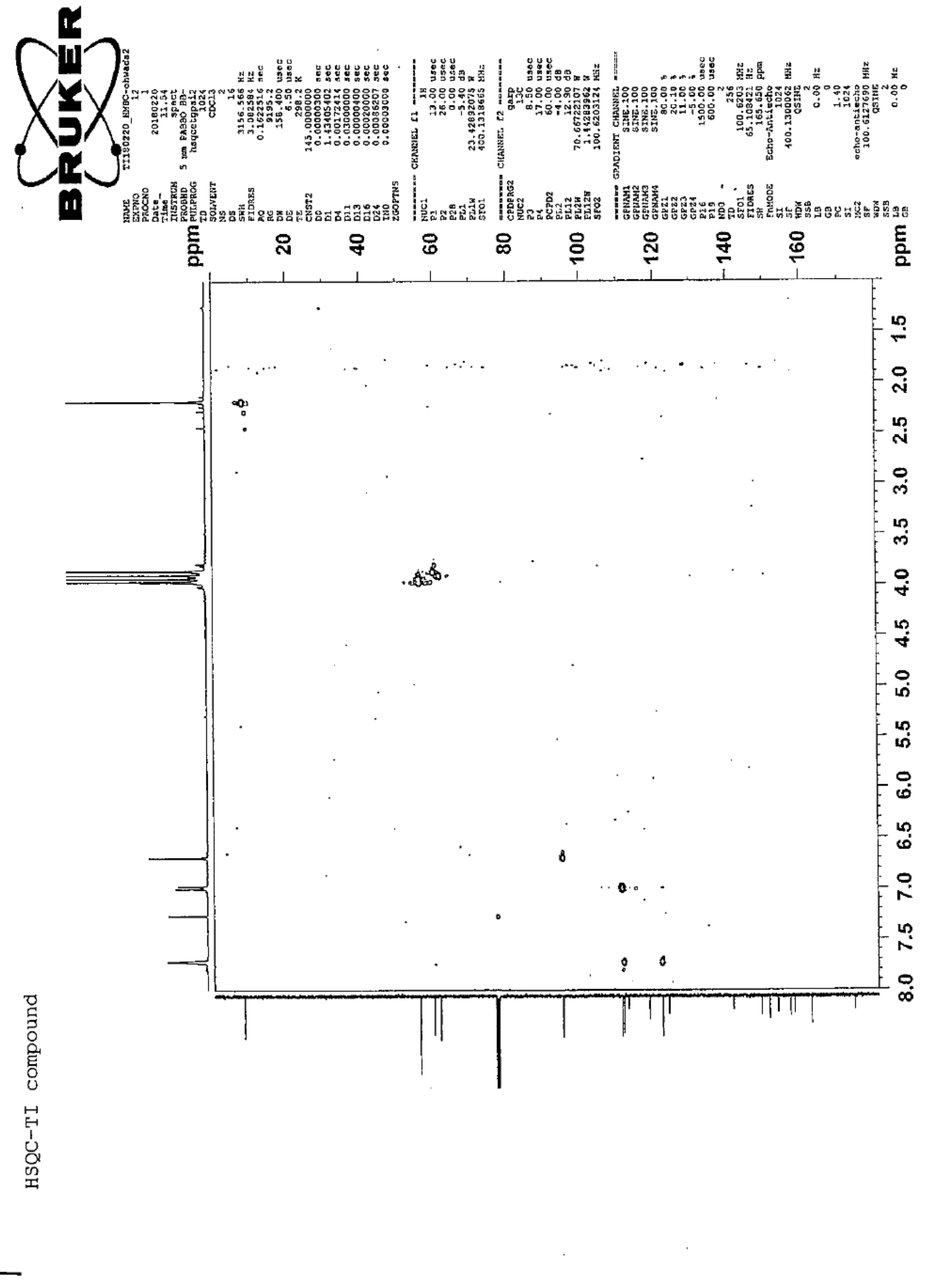

Fig. S12. HMBC of 6-methylquercetin pentamethyl ether **5**

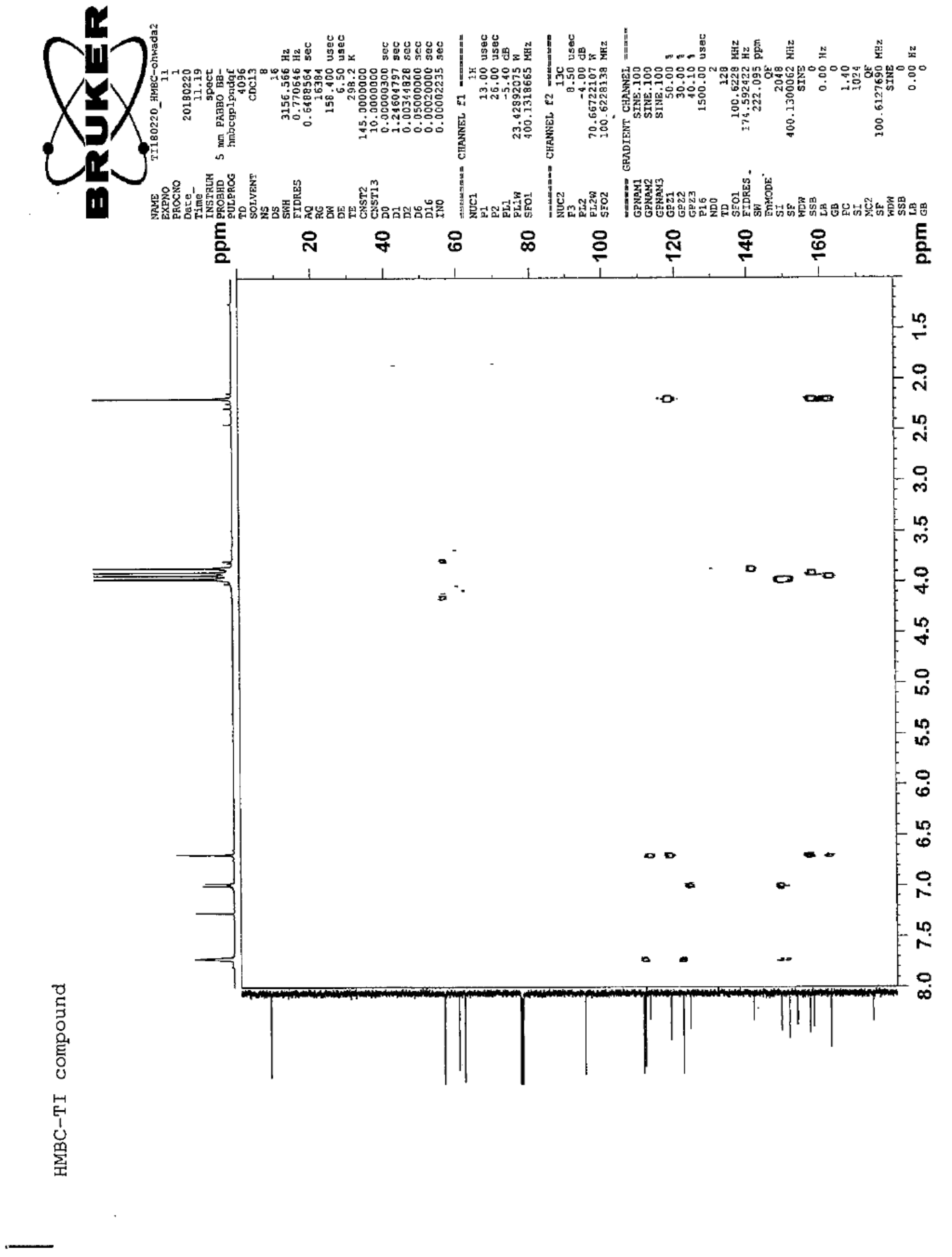

Supplement: File 2 — NMR charts of methylated products 1, 3, 4 and 5. [file Beilstein_J_Org_Chem-14-3112-s002.pdf]
